# Convenient Synthesis of Functionalized Cyclopropa[*c*]coumarin-1a-carboxylates

**DOI:** 10.3390/molecules24010057

**Published:** 2018-12-24

**Authors:** Olga A. Ivanova, Vladimir A. Andronov, Irina I. Levina, Alexey O. Chagarovskiy, Leonid G. Voskressensky, Igor V. Trushkov

**Affiliations:** 1 Department of Chemistry, M. V. Lomonosov Moscow State University, Leninskie gory 1-3, Moscow 119991, Russia; andronov.vlad@gmail.com; 2 N. M. Emanuel Institute of Biochemical Physics, Russia Academy of Sciences, Kosygina 4, Moscow 119334, Russia; iilevina@inbox.ru; 3 Laboratory of Chemical Synthesis, Dmitry Rogachev National Research Center of Pediatric Hematology, Oncology and Immunology, Samory Mashela 1, Moscow 117997, Russia; alex.chagarovskiy@gmail.com; 4 Faculty of Science, RUDN University, Miklukho-Maklaya 6, Moscow 117198, Russia; lvoskressensky@sci.pfu.edu.ru

**Keywords:** donor-acceptor cyclopropanes, coumarins, heterocycles

## Abstract

A simple method has been developed for the synthesis of cyclopropa[*c*]coumarins, which belong to the donor-acceptor cyclopropane family and, therefore, are promising substrates for the preparation of chromene-based fine chemicals. The method, based on the acetic acid-induced intramolecular transesterification of 2-arylcyclopropane-1,1-dicarboxylates, was found to be efficient for substrates containing hydroxy group directly attached to the aromatic ring.

## 1. Introduction

The longstanding interest to coumarin derivatives stems from their wide distribution in Nature and a broad spectrum of bioactivity, including anticoagulant, anticonvulsant, antidepressant, anti-HIV, antiinflammatory, antimicrobial, antioxidative, antituberculosis, antitumor activities, etc. [1,2,3,4,5,6,7,8,9,10,11]. Coumarin derivatives are used in medicinal practice for the treatment of dementia [12], varicose veins [13], hemorrhoids [13], etc. [14]; they are applied as rodenticides [15] and as fluorophores in cell biology [16]. The high value of these compounds has stimulated their intensive investigation, i.e. the synthesis of novel coumarin derivatives, the study of their transformations and screening of their bioactivity, 3- and 4-substituted coumarins as well as their 3,4-dihydro derivatives being the most interesting substrates for the medicinal applications.

**Scheme 1:**
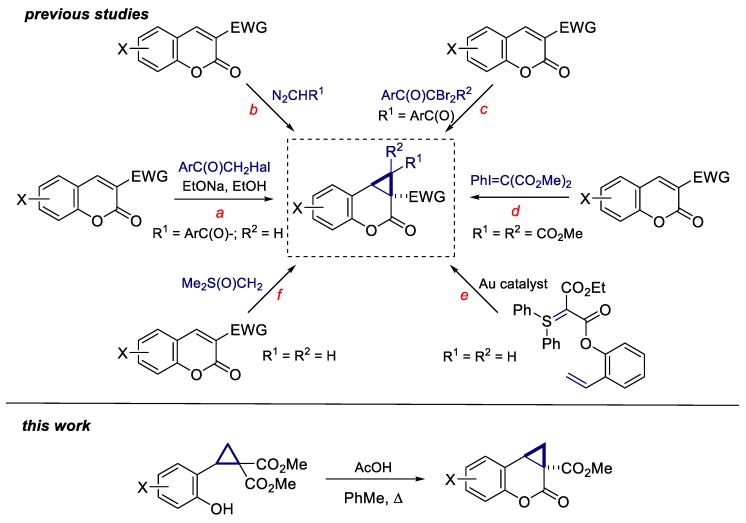
Syntheses of cyclopropa[*c*]coumarins.

Cyclopropa[*c*]coumarins, being the members of the donor-acceptor cyclopropanes subclass, are highly potent substrates for the preparation of a large diversity of functionalized coumarin derivatives due to a rich reactivity of the three-membered ring. Even though the first cyclopropanation of 3-acylcoumarins and coumarin-3-carboxylates with α-haloketones was described by Widman exactly 100 years ago (Scheme 1a) [17,18], cyclopropacoumarins remain poorly investigated due to the absence of efficient methods for their synthesis. Since Widman’s study, a number of cyclopropa[*c*]coumarins bearing diverse acceptor substituents at both C(1) and C(1a) atoms have been obtained by the related cyclopropanations under phase transfer catalysis (Scheme 1a) [19] or using diazoketones (Scheme 1b) [20,21,22]. Nevertheless, the application of diazoketones usually resulted in the formation of complex mixtures and low yields of target products. Moreover, 1,1,1a-substituted cyclopropa[*c*]coumarins were synthesized in reasonable yields by the treatment of coumarin-3-carboxylates and -carboxamides with α,α-dibromoketones and zinc (Scheme 1c) [23,24,25], trichloroacetic acid [26], phenyliodonium (Scheme 1d) [27] and diphenylsulfonium ylides (Scheme 1e) [28].

Oppositely, the reaction of much more reactive dimethylsulfoxonium methylide [29,30] with 3-acylcoumarins, coumarin-3-carboxylates, the corresponding nitriles as well as sulfones under the typical conditions of Corey-Chaykovsky cyclopropanation was found to afford cyclopenta[*b*]-benzofuran derivatives as a result of the fast secondary reaction of the initially formed cyclopropa[*c*]coumarins with a second equivalent of the Corey ylide [31,32,33]. The target cyclopropa[*c*]coumarins were obtained in low-to-moderate yields when the reaction was performed at 0 °C (for 3-ethoxycarbonyl-, 3-pivaloyl- and 3-cyanocoumarins) or at −40 °C (for 3-acetyl and 3-benzoyl derivatives) (Scheme 1f) [31]. Very recently it was proposed to perform this cyclopropanation using very slow addition of the Corey ylide solution (5 mL for 6 h) to the corresponding coumarin derivative, however, product yields were not given [34,35]. Therefore, the development of efficient procedure for the synthesis of 1-unsubstituted cyclopropa[*c*]coumarins remains a challenging task for organic chemists. Herein, we describe a new general approach to cyclopropa[*c*]coumarins based on the use of 2-(2-hydroxyphenyl)cyclopropane-1,1-dicarboxylates (Scheme 1) which can be easily obtained from the corresponding salicylic aldehydes [36,37]. 

## 2. Results and Discussion

We started our research with a search for the optimal conditions for the transesterification of 2-hydroxy- and 2-(methoxymethoxy)phenyl-substituted cyclopropane-1,1-dicarboxylates **1a**,**b** as model substrates using their treatment with base (K_2_CO_3_ or *N*,*N*-diisopropylethylamine, DIPEA) or Brønsted acid (Table 1). We have found that the highest yield of the target methyl cyclopropa[*c*]coumarin-3-carboxylate **2a** was obtained when toluene solution of cyclopropane **1a** was heated under reflux with 2 equiv of acetic acid (Table 1, entry 8). The use of stronger acids such as trifluoroacetic acid (TFA) or *p*-toluenesulfonic acid (TsOH) resulted in the formation of complex mixture of products; any attempts to isolate **2a** were unsuccessful. Base-induced reaction produced the desired product **2a** in low yield only.

**Scheme 2:**
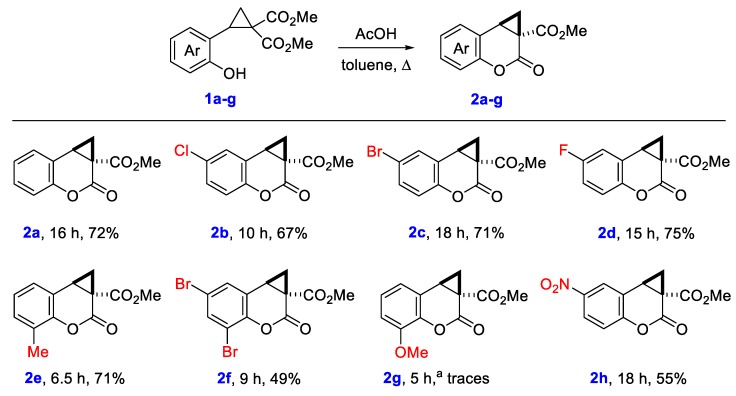
Synthesis of cyclopropa[*c*]coumarins **2a**–**h**. *^a^* In chlorobenzene.

With the optimized reaction conditions in hand, we studied the scope of this transesterification and found that diverse substituents in the benzene ring (halogens, alkyl or nitro group) are tolerant to the reaction conditions (Scheme 2). Oppositely, the reaction of 2-hydroxy-3-methoxyphenyl-substituted cyclopropane **1g** produced only trace amounts of the corresponding cyclopropa[*c*]coumarin **2g**.

**Scheme 3:**
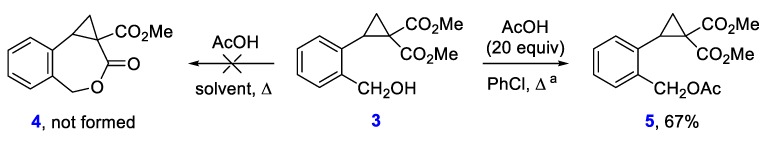
The attempt to synthesize benzoxepane **4**. *^a^* Substrate concentration: 0.04 M.

To extend the scope of the reaction, we have studied reactions of cyclopropane **3** with a 2-(hydroxymethyl)phenyl group as a donor and its analogue **8** bearing a 3-hydroxymethyl-4-indolyl substituent. However, in both cases we failed to obtain the corresponding products of intramolecular esterification. Thus, the heating of **3** with 2 equiv of acetic acid in both toluene and chlorobenzene did not produce the desired oxepanone **4** at all. When a large excess of acetic acid was applied, benzyl acetate **5** was obtained as a single low-molecular-weight product (Scheme 3). The use of a stronger acid led to the formation of complex mixtures containing predominantly products of the three-membered ring opening.

**Scheme 4:**
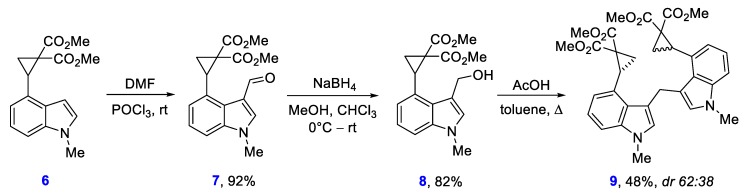
Synthesis and acetic acid-induced dimerization of indolylmethanol **8**.

Meanwhile, indolyl-substituted cyclopropane **8**, which was obtained in two steps from the reported cyclopropane **6** [38], under heating with acetic acid afforded bis(indolyl)methane **9** (Scheme 4). Similar AcOH-induced transformations of 3-indolylmethanols to bis(indolyl)methanes was previously described [39,40,41]. Meanwhile, the presence of highly reactive donor-acceptor cyclopropane functionality in the starting alcohol **8** and two such moieties in the product **9** provides non-trivial nature this process.

**Scheme 5:**
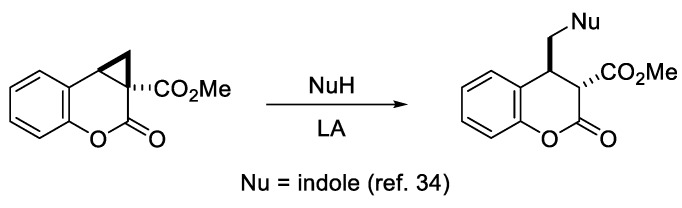
Nucleophilic ring opening of cyclopropa[*c*]coumarins.

The obtained cyclopropa[*c*]coumarins **2** are potent substrates for the synthesis of diverse coumarin derivatives (Scheme 5). Thus, it was recently shown that such cyclopropacoumarins undergo Ni(ClO_4_)_2_-catalyzed nucleophilic ring opening under treatment with indole affording 4-(indolylmethyl)chroman-2-one-3-carboxylates [34].

**Scheme 6:**
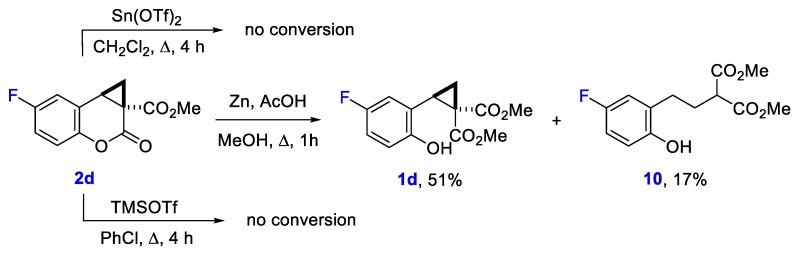
Attempts of a ring enlargement of cyclopropa[*c*]coumarin **2d**.

Nevertheless, our attempts to transform compounds **2** to the corresponding benzoxepan derivatives via their reduction with Zn/AcOH system [42] or Lewis acid-induced isomerization [43] were unsuccessful. Unexpectedly, the treatment of cyclopropacoumarin **2d** with zinc and acetic acid in methanol did not afford the products of three-membered ring reduction. Instead, it led to the methanolysis of the lactone moiety in **2d** producing cyclopropane **1d** and a small amount of acyclic product **10** formed by the reduction of **1d** (Scheme 6). Moreover, this cyclopropacoumarin remains intact under heating with both tin(II) triflate in dichloromethane and trimethylsilyl triflate in chlorobenzene for several hours. The absence of benzoxepane derivative in the reaction mixtures together with the aforementioned literature data on the nucleophilic attack of cyclopa[*c*]coumarins at the CH_2_ atom [34] demonstrate the decelerating effect of annulation on the reactivity of the C-C bond between atoms connected to donor and acceptor substituents. The effect of the annulated ring nature on the reactivity of donor-acceptor cyclopropanes deserve, evidently, a careful study.

## 3. Experimental

### 3.1. General Information

The structures of synthesized compounds were elucidated with the aid of 1D NMR (^1^H, ^13^C) and 2D NMR (HSQC and HMBC ^1^H-^13^C) spectroscopy. NMR spectra were acquired on Avance 500 (Bruker, Billerica, MA, USA) and 400-MR (Agilent, Santa Clara, CA, USA) spectrometers at room temperature; the chemical shifts *δ* were measured in ppm with respect to solvent (^1^H: CDCl_3_, *δ* = 7.27 ppm; ^13^C: CDCl_3_, *δ* = 77.0). Splitting patterns are designated as s, singlet; d, doublet; m, multiplet; dd, double doublet; br., broad. Coupling constants (*J*) are in Hertz. Infrared spectra were recorded on an Infralum FT-801 spectrometer (Simex, Novosibirsk, Russia). High resolution and accurate mass measurements were carried out using a micrOTOF-Q^TM^ ESI-TOF (Electrospray Ionization/Time of Flight, Bruker, Billerica, MA, USA), LTQ Orbitrap (Thermo Fischer Scientific, Waltham, MA, USA) mass spectrometers and a Triple TOF 5600+ instrument (AB Sciex, Darmstadt, Germany) using ESI modes. Elemental analyses were performed with an EA-1108 CHNS elemental analyser instrument (Fisons, Ipswich, UK). Melting points (mp) are uncorrected and were measured on a 9100 capillary melting point apparatus (Electrothermal, Stone, UK). Analytical thin layer chromatography (TLC) was carried out with silica gel plates (silica gel 60, F_254_, supported on aluminium); visualization was done using a UV lamp (365 nm). Column chromatography was performed on silica gel 60 (230–400 mesh, Merck, Darmstadt, Germany). All reactions were carried out using freshly distilled and dry solvents. Dimethyl 2-(2-hydroxyaryl)cyclopropane-1,1-dicarboxylate was synthesized by the published procedure [36,37]. Commercial reagents employed in the synthesis were analytical grade, obtained from Aldrich (St. Louis, MI, USA) or Alfa Aesar (Ward Hill, MO, USA). Cyclopropanes **3** and **6** were obtained by the reported procedures [36,38]. The ^1^H NMR, ^13^C NMR for synthesized compounds as well as 2D (HSQC and HMBC) NMR spectra for selected compounds are available in the Supplementary Material.

### 3.2. General Procedure for the Synthesis of Cyclopropa[c]coumarins

**Figure 1:**
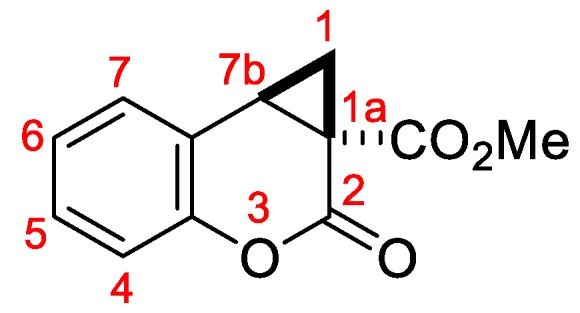
Atom numbering in compounds **2**.

A toluene solution of cyclopropane **1** (0.03 or 0.04 M, 1 equiv) and glacial acetic acid (2 equiv) was heated for the specified time under reflux or using backflow condenser without cooling with water providing slow removal of the formed methanol. When **1** was completely converted (TLC control), the reaction mixture was cooled, diluted with ether (10 mL), washed with saturated NaHCO_3_ solution (3 × 15 mL), dried with Na_2_SO_4_ and concentrated in vacuo. The resulting residue was purified by flash chromatography on silica gel (eluent: 10–50% ethyl acetate in petroleum ether) to afford the target cyclopropacoumarin **2** (Figure 1).

*Methyl (1aRS,7bRS)-2-oxo-1,7b-dihydrocyclopropa[c]chromene-1a(2H)-carboxylate* (**2a**). Dimethyl 2- (2-hydroxyphenyl)cyclopropane-1,1-dicarboxylate (**1a**, 120 mg, 0.48 mmol), AcOH (58 mg, 55 µL, 0.96 mmol), toluene (12 mL), reflux, 16 h. *R_f_* = 0.55 (ethyl acetate:petroleum ether 1:2). Yield 75 mg (72%); colorless solid; mp = 98–100 °C (lit. 100–102 °C [34,35]; oil [37]). Spectral data are consistent with the reported ones [34,35].

*Methyl (1aRS,7bRS)-6-chloro-2-oxo-1,7b-dihydrocyclopropa[c]chromene-1a(2H)-carboxylate* (**2b**). Dimethyl 2-(5-chloro-2-hydroxyphenyl)cyclopropane-1,1-dicarboxylate (**1b**) (120 mg, 0.42 mmol), AcOH (51 mg, 48 µL, 0.84 mmol), toluene (12 mL), reflux, 10 h. *R_f_* = 0.31 (ethyl acetate:petroleum ether 1:4). Yield 71 mg (67%); colorless solid; mp = 111–112 °C (lit. 119–121 °C [34]). ^1^H-NMR (CDCl_3_, 500 MHz) *δ* = 1.41 (dd, ^2^*J* = 5.2 Hz, ^3^*J* = 6.4 Hz, 1H, CH_2_), 2.50 (dd, ^2^*J* = 5.2 Hz, ^3^*J* =9.2 Hz, 1H, CH_2_), 2.89 (dd, ^3^*J* = 9.2 Hz, ^3^*J* = 6.4 Hz, 1H, CH), 3.86 (s, 3H, CH_3_O), 7.00 (d, ^3^*J* = 8.7 Hz, 1H, Ar), 7.24 (dd, ^3^*J* = 8.7 Hz, ^4^*J* = 2.3 Hz, 1H, Ar), 7.37 (d, ^4^*J* = 2.3 Hz, 1H, Ar). ^13^C-NMR (CDCl_3_, 125 MHz) *δ* = 21.1 (CH_2_), 28.7 (CH), 29.7 (C), 53.4 (CH_3_O), 118.6 (CH, Ar), 121.7 (C, Ar), 127.6 (CH, Ar), 128.6 (CH, Ar), 129.8 (C, Ar), 148.0 (C, Ar), 161.7 (CO), 167.7 (*C*O_2_Me). IR (сm^−1^) 3110, 3066, 2950, 2955, 2922, 2853, 1763, 1723, 1484, 1435, 1369, 1319, 1283, 1259, 1107, 1085, 1052, 946. HRMS ESI-TOF: *m/z* = 253.0265 [M + H]^+^ (253.0262 сalcd for C_12_H_10_ClO_4_).

*Methyl (1aRS,7bRS)-6-bromo-2-oxo-1,7b-dihydrocyclopropa[c]chromene-1a(2H)-carboxylate* (**2c**). Dimethyl 2-(5-bromo-2-hydroxyphenyl)cyclopropane-1,1-dicarboxylate (**1c**, 200 mg, 0.61 mmol), AcOH (73 mg, 70 µL, 1.2 mmol), toluene (15 mL), reflux, 18 h. *R_f_* = 0.70 (ethyl acetate:petroleum ether 1:4). Yield 126 mg (71%); colorless solid; mp = 114–115 °C (lit. 105–107 °C [34]). ^1^H-NMR (CDCl_3_, 500 MHz) *δ* = 1.40 (dd, ^2^*J* = 5.1 Hz, ^3^*J* = 6.4 Hz, 1H, CH_2_), 2.21 (dd, ^2^*J* = 5.1 Hz, ^3^*J* = 9.2 Hz, 1H, CH_2_), 2.89 (dd, ^3^*J* = 9.2 Hz, ^3^*J* = 6.4 Hz, 1H, CH), 3.85 (s, 3H, CH_3_O), 6.92 (d, ^3^*J* = 8.7 Hz, 1H, Ar), 7.38 (dd, ^3^*J* = 8.7 Hz, ^4^*J* = 2.3 Hz, 1H, Ar), 7.50 (d, ^4^*J* = 2.3 Hz, 1H, Ar). ^13^C-NMR (CDCl_3_, 125 MHz) *δ* = 21.1 (CH_2_), 28.3 (C), 28.5 (CH), 53.3 (CH_3_O), 117.0 (C, Ar), 118.8 (CH, Ar), 122.1 (C, Ar), 130.4 (CH, Ar), 131.5 (CH, Ar), 148.5 (C, Ar), 161.5 (CO), 167.6 (*C*O_2_Me). IR (сm^−1^) 3256, 2956, 1762, 1673, 1498, 1440, 1415, 1352, 1276, 1196, 1154, 1108, 1074. HRMS ESI-TOF: *m/z* = 296.9757 [M + H]^+^ (296.9757 сalcd for C_12_H_10_BrO_4_).

*Methyl (1aRS,7bRS)-6-fluoro-2-oxo-1,7b-dihydrocyclopropa[c]chromene-1a(2H)-carboxylate* (**2d**). Dimethyl 2-(5-fluoro-2-hydroxyphenyl)cyclopropane-1,1-dicarboxylate (**1d**, 200 mg, 0.75 mmol), AcOH (90 mg, 85 µL, 1.49 mmol), toluene (11 mL), boiling with backflow condenser, 8 h; then an extra portion of AcOH (90 mg, 85 µL, 1.49 mmol), reflux, 7 h. *R_f_* = 0.69 (ethyl acetate:petroleum ether 1:2). Yield 150 mg (75%); colorless solid; mp = 90–91 °C (lit. 93–95 °C [34]). ^1^H-NMR (CDCl_3_, 400 MHz) *δ* = 1.40 (dd, ^2^*J* = 5.1 Hz, ^3^*J* = 6.3 Hz, 1H, CH_2_), 2.59 (dd, ^2^*J* = 5.1 Hz, ^3^*J* =9.0 Hz, 1H, CH_2_), 2.88 (dd, ^3^*J* = 9.0 Hz, ^3^*J* = 6.3 Hz, 1H, CH), 3.84 (s, 3H, CH_3_O), 6.93–7.02 (m, 2H, Ar), 7.07 (dd, ^3^*J* = 8.2 Hz, ^4^*J*_HF_ = 3.0 Hz, 1H, Ar). ^13^C- NMR (CDCl_3_, 100 MHz) *δ* = 21.2 (CH_2_), 28.3 (C), 29.0 (CH), 53.5 (CH_3_O), 114.5 (d, ^2^*J*_CF_ = 24 Hz, CH, Ar), 115.5 (d, ^2^*J*_CF_ = 24 Hz, CH, Ar), 118.7 (d, ^3^*J*_CF_ = 9 Hz, CH, Ar), 121.7 (d, ^3^*J*_CF_ = 8 Hz, C, Ar), 145.6 (C, Ar), 158.5 (d, ^1^*J*_CF_ = 243 Hz, C, Ar), 162.1 (CO), 167.9 (*C*O_2_Me). IR (сm^−1^) 1759, 1724, 1579, 1496, 1441, 1375, 1325, 1288, 1250, 1197, 1149, 1107, 1057, 987, 966, 931. HRMS ESI-TOF: *m/z* = 237.0555 [M + H]^+^ (237.0558 сalcd for C_12_H_10_ FO_4_). 

*Methyl (1aRS,7bRS)-4-methyl-2-oxo-1,7b-dihydrocyclopropa[c]chromene-1a(2H)-carboxylate* (**2e**). Dimethyl 2-(3-methyl-2-hydroxyphenyl)cyclopropane-1,1-dicarboxylate (**1e**, 180 mg, 0.64 mmol), AcOH (158 mg, 160 µL, 2.6 mmol), toluene (12 mL), reflux, 10 h. *R_f_* = 0.75 (ethyl acetate:petroleum ether 1:2). Yield 113 mg (76%); colorless solid; mp = 82–83 °C. ^1^H-NMR (CDCl_3_, 500 MHz) *δ* = 1.37 (dd, ^2^*J* = 4.9 Hz, ^3^*J* = 6.7 Hz, 1H, CH_2_), 2.30 (s, 3H, CH_3_), 2.46 (dd, ^2^*J* = 4.9 Hz, ^3^*J* =9.2 Hz, 1H, CH_2_), 2.90 (dd, ^3^*J* = 9.2 Hz, ^3^*J* = 6.7 Hz, 1H, CH), 3.84 (s, 3H, CH_3_O), 7.03 (dd, ^3^*J* = 7.6 Hz, ^3^*J* = 7.4 Hz, 1H, Ar), 7.11 (br. d, ^3^*J* = 7.6 Hz, 1H, Ar), 7.18 (br. d, ^3^*J* = 7.4 Hz, 1H, Ar). ^13^C-NMR (CDCl_3_, 125 MHz) *δ* = 15.6 (CH_3_), 20.9 (CH_2_), 28.6 (C), 29.3 (CH), 53.1 (CH_3_O), 119.6 (C, Ar), 124.0 (CH, Ar), 125.2 (CH, Ar), 126.5 (C, Ar), 130.0 (CH, Ar), 147.7 (C, Ar), 162.4 (CO), 168.1 (*C*O_2_Me). IR (сm^−1^) 3106, 3040, 2957, 2924, 2853, 1746, 1730, 1471, 1434, 1395, 1327, 1292, 1230, 1192, 1116, 1098, 1057, 1039, 946, 925. HRMS ESI-TOF: *m/z* = 233.0810 [M + H]^+^ (233.0808 сalcd for C_13_H_13_O_4_).

*Methyl (1aRS,7bRS)-4,6-dibromo-2-oxo-1,7b-dihydrocyclopropa[c]chromene-1a(2H)-carboxylate* (**2f**). Dimethyl 2-(3,5-dibromo-2-hydroxyphenyl)cyclopropane-1,1-dicarboxylate (**1f**, 200 mg, 0.49 mmol), AcOH (30 mg, 28 µL, 0.49 mmol), toluene (11 mL), boiling with backflow condenser, 5 h, then an extra portion of AcOH (60 mg, 56 µL, 0.98 mmol), reflux, 4 h. *R_f_* = 0.52 (ethyl acetate:petroleum ether 1:4). Yield 90 mg (49%); colorless solid; mp = 77–78 °C. ^1^H-NMR (CDCl_3_, 500 MHz) *δ* = 1.45 (dd, ^2^*J* = 5.2 Hz, ^3^*J* = 6.4 Hz, 1H, CH_2_), 2.51 (dd, ^2^*J* = 5.2 Hz, ^3^*J* =9.2 Hz, 1H, CH_2_), 2.90 (dd, ^3^*J* = 9.2 Hz, ^3^*J* = 6.4 Hz, 1H, CH), 3.86 (s, 3H, CH_3_O), 7.47 (d, ^4^*J* = 2.3 Hz, 1H, Ar), 7.67 (d, ^4^*J* = 2.3 Hz, 1H, Ar). ^13^C-NMR (CDCl_3_, 125 MHz) *δ* = 21.1 (CH_2_), 28.8 (C), 29.0 (CH), 53.7 (CH_3_O), 112.0 (C, Ar), 117.2 (C, Ar), 123.5 (C, Ar), 129.8 (CH, Ar), 134.9 (CH, Ar), 146.0 (C, Ar), 160.6 (CO), 167.5 (*C*O_2_Me). IR (film, сm^−1^) 3447, 3078, 3005, 2954, 2924, 2851, 1777, 1725, 1453, 1439, 1368, 1324, 1239, 1207, 1071, 1045, 991, 985. HRMS ESI-TOF: *m/z* = 374.8856 [M + H]^+^ (374.8862 сalcd for C_12_H_9_Br_2_O_4_).

*Methyl (1aRS,7bRS)-6-nitro-2-oxo-1,7b-dihydrocyclopropa[c]chromene-1a(2H)-carboxylate* (**2h**). Dimethyl 2-(5-nitro-2-hydroxyphenyl)cyclopropane-1,1-dicarboxylate (**1h**, 200 mg, 0.68 mmol), AcOH (81 mg, 78 µL, 1.35 mmol), toluene (17 mL), boiling with backflow condenser, 7 h, then an extra portion of AcOH (81 mg, 78 µL, 1.35 mmol), reflux, 10 h. *R_f_* = 0.63 (ethyl acetate:petroleum ether 1:4). Yield 97 mg (55%); colorless solid; mp = 146–147 °C. ^1^H-NMR (CDCl_3_, 500 MHz) *δ* = 1.49 (dd, ^2^*J* = 5.3 Hz, ^3^*J* = 6.4 Hz, 1H, CH_2_), 2.59 (dd, ^2^*J* = 5.3 Hz, ^3^*J* =9.2 Hz, 1H, CH_2_), 3.06 (dd, ^3^*J* = 9.2 Hz, ^3^*J* = 6.4 Hz, 1H, CH), 3.88 (s, 3H, CH_3_O), 7.19 (d, ^3^*J* = 9.0 Hz, 1H, Ar), 8.18 (dd, ^3^*J* = 9.0 Hz, ^4^*J* = 2.8 Hz, 1H, Ar), 8.33 (br. d, ^4^*J* = 2.8 Hz, 1H, Ar). ^13^C-NMR (CDCl_3_, 125 MHz) *δ* = 21.2 (CH_2_), 28.3 (C), 28.4 (CH), 53.5 (CH_3_O), 118.2 (CH, Ar), 121.3 (C, Ar), 123.8 (CH, Ar), 124.3 (CH, Ar), 144.2 (C, Ar), 153.7 (C, Ar), 160.6 (CO), 167.2 (*C*O_2_Me). IR (film, сm^−1^) 3117, 2957, 2923, 2854, 1778, 1730, 1591, 1527, 1492, 1443, 1337, 1316, 1252, 1128, 1107, 1048, 979. HRMS ESI-TOF: *m/z* = 264.0502 [M + H]^+^ (264.0503 сalcd for C_12_H_10_NO_6_). 

*Dimethyl 2-[2-(acetoxymethyl)phenyl]cyclopropane-1,1-dicarboxylate* (**5**). A solution of cyclopropane **3** (190 mg, 0.72 mmol) and glacial acetic acid (1.6 mL, 28 mmol, 40 equiv.) in chlorobenzene (14 mL) was refluxed for 16 h, cooled, diluted with CH_2_Cl_2_ (10 mL), washed with saturated NaHCO_3_ solution (3 × 15 mL), dried with Na_2_SO_4_ and concentrated in vacuo. The resulting residue was purified by flash chromatography on silica gel (eluent: 10–30% ethyl acetate in petroleum ether) to afford acetate **4** as colorless oil. *R_f_* = 0.57 (ethyl acetate:petroleum ether 1:3). Yield 148 mg (67%). ^1^H-NMR (CDCl_3_, 400 MHz) *δ* = 1.74 (dd, ^2^*J* = 5.2 Hz, ^3^*J* = 9.2 Hz, 1H, CH_2_), 2.07 (s, 3H, CH_3_CO), 2.28 (dd, ^2^*J* = 5.2 Hz, ^3^*J* = 8.2 Hz, 1H, CH_2_), 3.28 (s, 3H, CH_3_O), 3.30 (dd, ^3^*J* = 9.2 Hz, ^3^*J* = 8.2 Hz, 1H, CH), 3.78 (s, 3H, CH_3_O), 5.12 (AB system, ^2^*J* = 12.7 Hz, 1H, CH_2_O), 5.27 (AB system, ^2^*J* = 12.7 Hz, 1H, CH_2_O), 7.07–7.10 (m, 1H, Ar), 7.23–7.27 (m, 2H, Ar), 7.30–7.33 (m, 1H, Ar). ^13^C-NMR (CDCl_3_, 100 MHz) *δ* = 18.2 (CH_2_), 20.8 (CH_3_), 29.9 (CH), 36.6 (C), 52.1 (CH_3_O), 52.8 (CH_3_O), 64.2 (CH_2_O), 127.5 (CH, Ar), 127.7 (CH, Ar), 128.1 (CH, Ar), 129.3 (CH, Ar), 133.1 (C, Ar), 136.2 (C, Ar), 166.7 (Me*C*O_2_), 169.8 (*C*O_2_Me), 170.7 (*C*O_2_Me). IR (сm^−1^) 3066, 3027, 3005, 2954, 2904, 2848, 2256, 1741, 1735, 1496, 1438, 1378, 1332, 1282, 1230, 1207, 1134, 1026, 968, 920. HRMS ESI-TOF: *m/z* = 307.1176 [M + Na]^+^ (307.1176 сalcd for C_16_H_19_O_6_).

*Dimethyl 2-(3-formyl-1-methyl-1H-indol-4-yl)cyclopropane-1,1-dicarboxylate* (**7**). DMF (1 mL) and POCl_3_ (0.3 mL, 3.2 mmol) were mixed at 10 °C under argon atmosphere. After 20 min to the resulting mixture cyclopropane **6** (840 mg, 2.9 mmol) in DMF (1 mL) was added. The obtained mixture was stirred at room temperature for 2 h and poured into ice-cold water (15 mL). The formed clear solution was treated with aq. NaOH (620 mg in 3 mL). Product was extracted with ethyl acetate. Combined organic fractions were washed with brine (4 × 10 mL) and dried with Na_2_SO_4_. Solvent was evaporated. Product was obtained as brown crystals (850 mg, 92%). Analytical sample was obtained by column chromatography on silica gel as yellow crystals. *R_f_* = 0.54 (ethyl acetate); mp 192–193 °C. ^1^H-NMR (CDCl_3_, 500 MHz) *δ* = 1.95 (dd, ^2^*J* = 5.0 Hz, ^3^*J* = 9.0 Hz, 1H, CH_2_), 2.40 (dd, ^2^*J* = 5.0 Hz, ^3^*J* = 8.1 Hz, 1H, CH_2_), 3.18 (s, 3H, CH_3_O), 3.87 (s, 6H, CH_3_N, CH_3_O), 4.01 (dd, ^3^*J* = 9.0 Hz, ^3^*J* = 8.1 Hz, 1H, CH), 7.07 (br.d, ^3^*J* = 7.3 Hz, 1H, Ind), 7.24 (dd, ^3^*J* = 8.2 Hz, ^3^*J* = 7.3 Hz, 1H, Ind), 7.31 (br.d, ^3^*J* = 8.2 Hz, 1H, Ind), 7.83 (s, 1H, Ind), 10.13 (s, 1H, CHO). ^13^C-NMR (CDCl_3_, 125 MHz) *δ* = 19.2 (CH_2_), 33.2 (CH), 33.8 (CH_3_N), 37.5 (C), 51.8 (CH_3_O), 52.7 (CH_3_O), 109.8 (CH, Ind), 119.1 (C, Ind), 122.1 (CH, Ind), 123.1 (CH, Ind), 125.9 (C, Ind), 128.3 (C, Ind), 138.1 (C, Ind), 138.5 (CH, Ind), 167.2 (*C*O_2_Me), 170.1 (*C*O_2_Me), 184.3 (CHO). IR (Nujol, сm^−1^): 2938, 2873, 1726, 1657, 1530, 1462, 1447, 1356, 1296, 1214, 1081, 1042. Anal. calcd for C_17_H_17_NO_5_: C, 64.75; H, 5.43; N, 4.44. Found: C, 64.59; H, 5.21; N, 4.35.

*Dimethyl 2-(3-hydroxymethyl-1-methyl-1H-indol-4-yl)cyclopropane-1,1-dicarboxylate* (**8**). To a stirred solution of cyclopropane **7** (381 mg, 1.2 mmol) in MeOH (1.2 mL) and CHCl_3_ (5 mL), NaBH_4_ (51 mg, 1.3 mmol) was added at 0 °C and stirred for 10 min. Then the mixture was allowed to warm to room temperature and stirred for 5 h until full conversion (TLC control). The solvent was removed in vacuo and the slurry was transferred to a separatory funnel with water and CH_2_Cl_2_. The product solution was extracted with CH_2_Cl_2_ (3 × 5 mL), the organic fractions were washed with water (3 × 5 mL) and dried with Na_2_SO_4_ affording the title compound. Yield 314 mg (82%). ^1^H-NMR (CDCl_3_, 500 MHz) *δ* = 1.84 (dd, ^2^*J* = 5.0 Hz, ^3^*J* = 8.9 Hz, 1H, CH_2_), 2.32 (dd, ^2^*J* = 5.0 Hz, ^3^*J* = 7.9 Hz, 1H, CH_2_), 3.05 (br. s, 1H, OH), 3.20 (s, 3H, CH_3_), 3.76 (s, 3H, CH_3_), 3.87 (s, 3H, CH_3_), 3.92 (dd, ^3^*J* = 8.9 Hz, ^3^*J* = 7.9 Hz, 1H, CH), 4.73 (d, ^2^*J* = 12.5 Hz, 1H, CH_2_), 4.97 (d, ^2^*J* = 12.5 Hz, 1H, CH_2_), 6.85 (br. d, ^3^*J* = 7.3 Hz, 1H, Ar), 7.13 (s, 1H, Ar), 7.14 (dd, ^3^*J* = 8.2 Hz, ^3^*J* = 7.3 Hz, 1H, Ar), 7.22 (br. d, ^3^*J* = 8.2 Hz, 1H, Ar). ^13^C-NMR (CDCl_3_, 125 MHz) *δ* = 18.3 (CH_2_), 31.4 (CH_3_N), 32.8 (CH), 37.4 (C), 52.1 (CH_3_O), 53.0 (CH_3_O), 57.1 (CH_2_O), 109.3 (CH, Ar), 115.0 (C, Ar), 118.6 (CH, Ar), 121.2 (CH, Ar), 126.1 (C, Ar), 126.9 (C, Ar), 129.9 (CH, Ar), 137.5 (C, Ar), 167.8 (*C*O_2_Me), 170.6 (*C*O_2_Me). IR (сm^−1^) 3476, 3022, 3003, 2951, 2925, 2855, 1726, 1674, 1455, 1437, 1334, 1281, 1210, 1132. HRMS ESI-TOF: *m/z* = 340.1159 [M + Na]^+^ (340.1155 сalcd for C_1__7_H_19_NNaO_5_).

*Dimethyl 2-[3-({4-[2,2-bis(methoxycarbonyl)cyclopropyl]-1-methyl-1H-indol-3-yl}methyl)-1-methyl-1H-indol-4-yl]cyclopropane-1,1-dicarboxylate* (**9**). Cyclopropane **8** (170 mg, 0.53 mmol), AcOH (32 mg, 30 L, 0.53 mmol), toluene (13 mL), boiling with backflow condenser, 6 h. *R_f_* = 0.63 (ethyl acetate:petroleum ether 1:2). Yield 75 mg (48%); colorless solid; mp = 79–80 °C. Mixture of diastereoisomers in the **A**:**B** ratio of 62:38; ^1^H-NMR (CDCl_3_, 500 MHz) for mixture of isomers: *δ* = 1.74 (dd, ^2^*J* = 5.0 Hz, ^3^*J* = 9.0 Hz, 2H, CH_2_, **A**), 1.83 (dd, ^2^*J* = 5.2 Hz, ^3^*J* = 9.4 Hz, 2H, CH_2_, **B**), 2.40 (dd, ^2^*J* = 5.2 Hz, ^3^*J* = 8.2 Hz, 2H, CH_2_, **B**), 2.45 (dd, ^2^*J* = 5.0 Hz, ^3^*J* = 8.4 Hz, 2H, CH_2_, **A**), 3.23 (s, 6H, CH_3_O, **B**), 3.29 (s, 6H, CH_3_O, **A**), 3.37 (s, 6H, CH_3_O, **B**), 3.56 3.29 (s, 6H, CH_3_O, **A**), 3.68 (s, 6H+6H, CH_3_N, **A**, **B**), 3.82 (dd, ^3^*J* = 9.0 Hz, ^3^*J* = 8.4 Hz, 2H, CH, **A**), 3.85 (dd, ^3^*J* = 9.4 Hz, ^3^*J* = 8.2 Hz, 2H, CH, **B**), 6.66 (broad signal, ν_1/2_ = 34 Hz, 2H, Ind, A), 6.69 (br. d, ^3^*J* = 7.3 Hz, 2H, Ind, **B**), 6.76 (br. s, 2H, Ind, **B**), 6.79 (br. d, ^3^*J* = 7.3 Hz, 2H, Ar, **A**), 7.07–7.13 (m, 2H + 2H, Ind, **A**, **B**), 7.18–7.21 (m, 2H + 2H, Ind, **A**, **B**). ^13^C-NMR (CDCl_3_, 125 MHz) for mixture of isomers: *δ* = 18.3 (2 × CH_2_, **A**), 19.7 (2 × CH_2_, **B**), 24.6 (CH_2_, **A**), 25.7 (CH_2_, **B**), 31.2 (2 × CH, **A**), 31.5 (2 × CH, **B**), 32.7 (2 × CH_3_N, **B**), 32.8 (2 × CH_3_N, **A**), 38.4 (2 × C, **A**), 38.7 (2 × C, **B**), 52.1 (2 × CH_3_O + 2 × CH_3_O, **A**, **B**), 52.2 (CH_3_O + CH_3_O, **A**, **B**), 52.4 (CH_3_O + CH_3_O, **A**, **B**), 108.9 (2 × CH, Ind, **B**), 109.0 (2 × CH, Ind, **A**), 114.9 (2 × C + 2 × C, Ind, **A**, **B**), 116.4 (2 × CH, Ind, **A**), 117.7 (2 × CH, A, **B**), 120.87 (2 × CH, Ar, **B**), 120.94 (2 × CH, Ind, **A**), 127.0 (2 × C, Ind, **A**), 127.4 (2 × C, Ind, **B**), 127.6 (2 × C, Ind, **B**), 127.7 (2 × C, Ind, **A**), 128.5 (2 × CH, Ind, **A**), 129.2 (2 × CH, Ind, **B**), 137.9 (2 × C + 2 × C, Ind, **A**, **B**), 167.27 (2 × *C*O_2_Me, **B**), 167.33 (2 × *C*O_2_Me, **A**), 169.8 (2 × *C*O_2_Me, **B**), 170.0 (2 × *C*O_2_Me, **A**). IR (сm^−1^) 3618, 3442, 3055, 3022, 2950, 2924, 2852, 2254, 1728, 1608, 1570, 1550, 1456, 1437, 1371, 1330, 1281, 1209, 1128, 1103, 910. HRMS ESI-TOF: *m/z* = 587.2390 [M + H]^+^ (587.2388 сalcd for C_33_H_35_N_2_O_8_). 

*Dimethyl 2-[2-(5-fluoro-2-hydroxyphenyl)ethyl]malonate* (**10**). To a solution of cyclopropane **1d** (236 mg, 1 mmol) in MeOH (10 mL) Zn dust (0.69 g, 0.01 mol) and glacial acetic acid (0.29 mL, 5 mmol) were added. The reaction mixture was refluxed for 1 h, cooled, remaining Zn was filtered off and washed with ethyl acetate (5 mL). Filtrates were combined and diluted with water (8 mL), MeOH was evaporated under reduced pressure and aqueous phase was extracted with ethyl acetate (3 × 10 mL). Combined organic fractions were washed with saturated NaHCO_3_ solution (2 × 5 mL), dried with Na_2_SO_4_ and concentrated in vacuo. The resulting residue was purified by flash chromatography on silica gel (eluent: 10–50% ethyl acetate in petroleum ether) to afford product **10** as a colorless liquid; *R_f_* = 0.63 (ethyl acetate:petroleum ether 1:2). ^1^H-NMR (CDCl_3_, 400 MHz) *δ* = 2.11–2.17 (m, 2H, CH_2_), 2.61–2.65 (m, 2H, CH_2_), 3.44 (dd, ^3^*J* = 7.6 Hz, ^3^*J* = 6.5 Hz, 1H, CH), 3.77 (s, 6H, 2 × CH_3_O), 6.10 (br. s, 1H, OH), 6.76–6.81 (m, 3H, Ar). ^13^C-NMR (CDCl_3_, 100 MHz) *δ* = 28.0 (CH_2_), 28.8 (CH_2_), 50.6 (CH), 53.0 (2 × CH_3_O), 114.1 (d, ^2^*J*_CF_ = 23 Hz, CH, Ar), 116.5 (d, ^2^*J*_CF_ = 23 Hz, CH, Ar), 116.9 (d, ^3^*J*_CF_ = 8 Hz, CH, Ar), 127.9 (d, ^3^*J*_CF_ = 7 Hz, C, Ar), 150.4 (C, Ar), 157.4 (d, ^1^*J*_CF_ = 238 Hz, C, Ar), 170.3 (2 × *C*O_2_Me). IR (сm^−1^) 3442, 3394, 2954, 1730, 1620, 1510, 1437, 1342, 1273, 1230, 1186, 1101, 1079, 1045. HRMS ESI-TOF: *m/z* = 271.0968 [M + H]^+^ (271.0976 сalcd for C_13_H_16_ FO_5_).

## 4. Conclusions

The simple method for the synthesis of cyclopropa[*c*]coumarins based on the acid-induced intramolecular transesterification of 2-(2-hydroxyaryl)cyclopropane-1,1-dicarboxylates has been developed. Various functional groups in the aromatic ring including alkyl, halogen and nitro functionalities were shown to be tolerant to the optimized reaction conditions. The proposed method was, however, found to be inefficient for the preparation of the corresponding lactones with a larger ring size. The investigation of the reactivity of the synthesized compounds is in progress.

## Figures and Tables

**Table 1:**
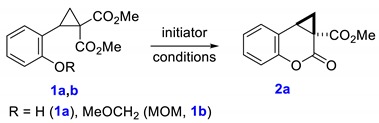
Optimization of reaction conditions for the intramolecular transesterification of model cyclopropanes **1a**,**b**.

Entry	Initiator (mol %)	R	Solvent	Temp (°C)	t (h)	Yield, % ^1^
1	K_2_CO_3_ (130) ^2^	H	DMSO	20	3	30
2	K_2_CO_3_ (130) ^2^	H	DMSO	20	25	30
3	DIPEA (120) ^3^	H	PhCl	100	5	45
4	TFA (110) ^3^	H	PhCl	reflux	7	- ^4^
5	TsOH (5) ^3^	H	CHCl_3_	reflux	5	- ^4^
6	AcOH (200) ^3^	H	PhMe	110 ^5^	8	30
7	AcOH (200) ^3^	H	PhMe	reflux	6	46
8	AcOH (200) ^3^	H	PhMe	reflux	9	61
9	AcOH (100) ^3^	H	PhCl	reflux	6.5	55 ^6^
**10**	**AcOH (200) ^3^**	**H**	**PhMe**	**reflux**	**16**	**72**
11	AcOH (200) ^3^	MOM	PhMe	reflux	16	-

^1^ Isolated yield. ^2^ 0.1 M solution of **1a**. ^3^ 0.03 M solution of **1a**,**b**. ^4^ Complex mixture of products. ^5^ Reaction was performed under microwave irradiation. ^6^ Dimethyl (2,3-dihydrobenzofuran-2-yl)malonate was also formed as side product. For details, see ref. [36].

## Supplementary Materials

Copies of ^1^H NMR and ^13^C NMR spectra for synthesized compounds as well as 2D (HSQC and HMBC) NMR spectra for selected compounds are available online.
