# Supplementary material for: Convenient Synthesis of Functionalized Cyclopropa[c]coumarin-1a-carboxylates
Source: Molecules. 2018 Dec 24;24(1):57. doi: 10.3390/molecules24010057 (PMC6337311; doi:10.3390/molecules24010057)

Convenient Synthesis of Functionalized Cyclopropa[*c*]coumarin-1a-carboxylates

Ivanova Olga A. [1,*], Andronov Vladimir A. [1], Levina Irina I. [2], Chagarovskiy Alexey O. [3], Voskressensky Leonid G. [4], Trushkov Igor V. [3,4,*]

[1] Department of Chemistry, M. V. Lomonosov Moscow State University, Leninskie gory 1-3, Moscow 119991, Russian Federation

[2] N. M. Emanuel Institute of Biochemical Physics, Russian Academy of Sciences, Kosygina 4, Moscow 119334, Russian Federation

[3] Laboratory of Chemical Synthesis, Dmitry Rogachev National Research Center of Pediatric Hematology, Oncology and Immunology, Samory Mashela 1, Moscow 117997, Russian Federation

[4] Faculty of Science, RUDN University, Miklukho-Maklaya 6, Moscow 117198, Russian Federation

Methyl (1a*RS*,7b*RS*)-6-chloro-2-oxo-1,7b-dihydrocyclopropa[*c*]chromene-1a(2*H*)-carboxylate (2b)

^{1}H NMR ($CDCl_3$, 500 MHz)

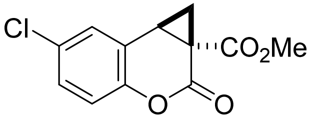

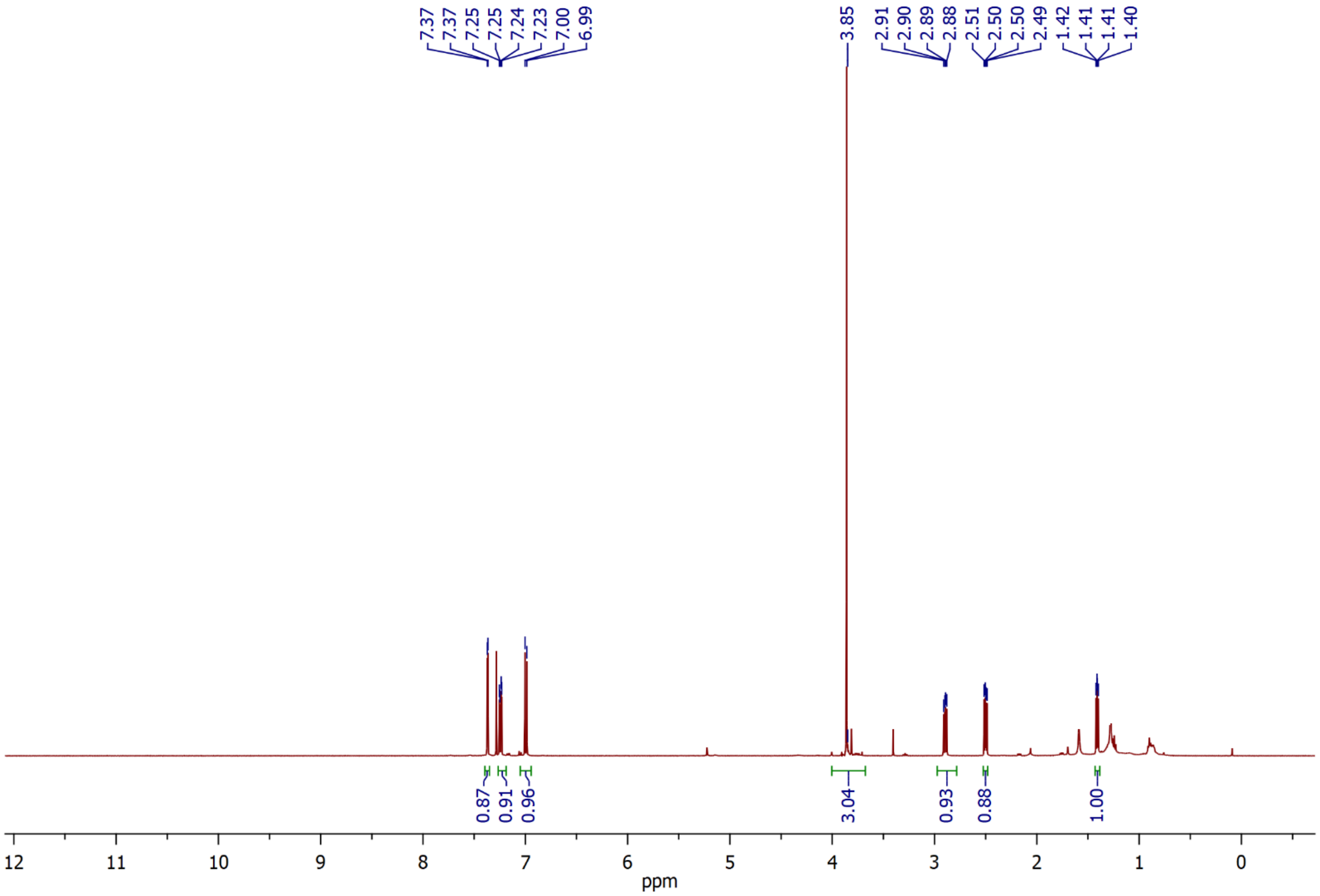

Methyl (1a*RS*,7b*RS*)-6-chloro-2-oxo-1,7b-dihydrocyclopropa[*c*]chromene-1a(2*H*)-carboxylate (2b)

^{13}C NMR ($CDCl_3$, 125 MHz)

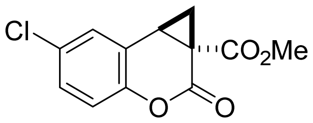

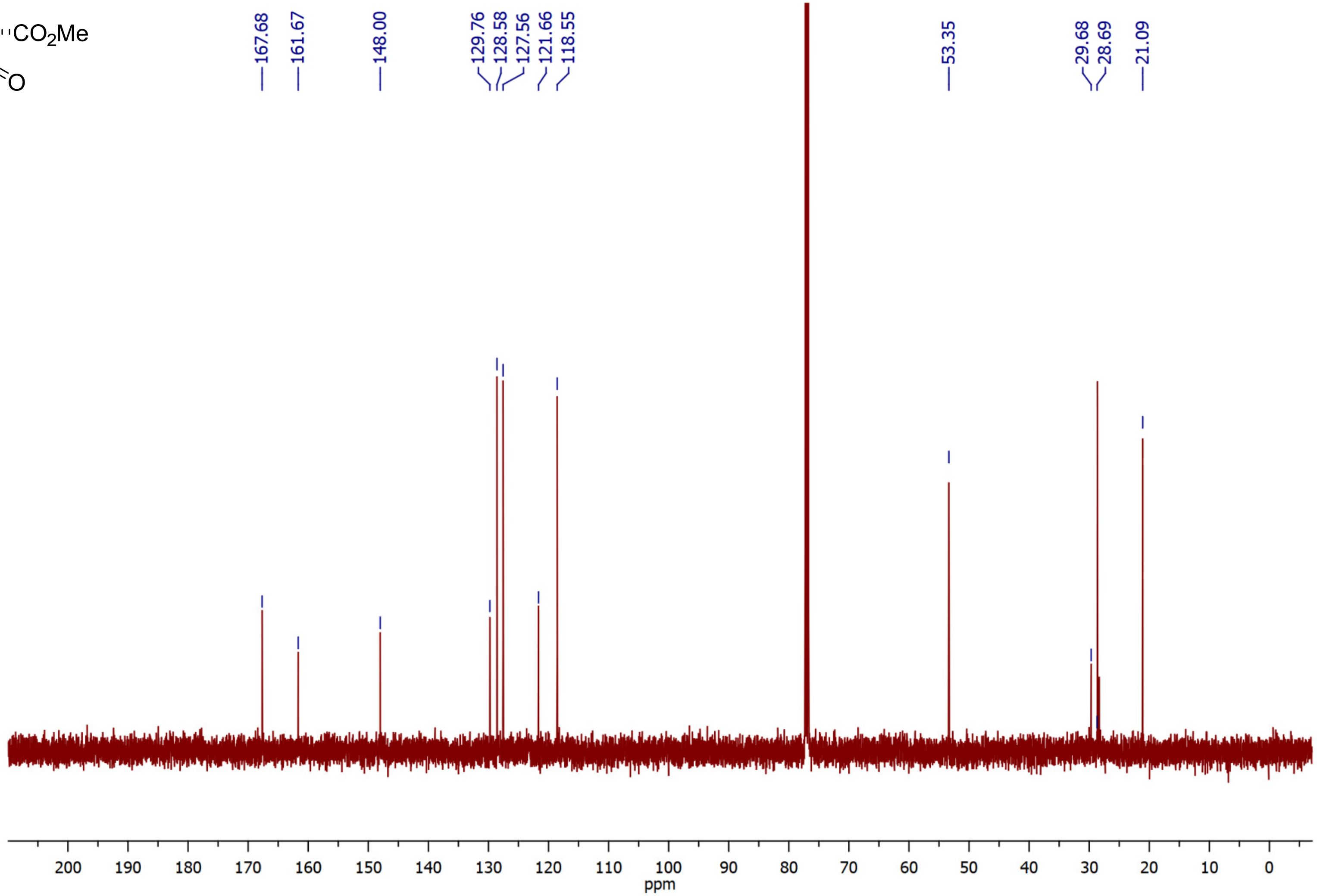

Methyl (1a*RS*,7b*RS*)-6-chloro-2-oxo-1,7b-dihydrocyclopropa[*c*]chromene-1a(2*H*)-carboxylate (2b)

HSQC ^{1}H-^{13}C ($CDCl_3$)

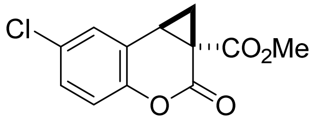

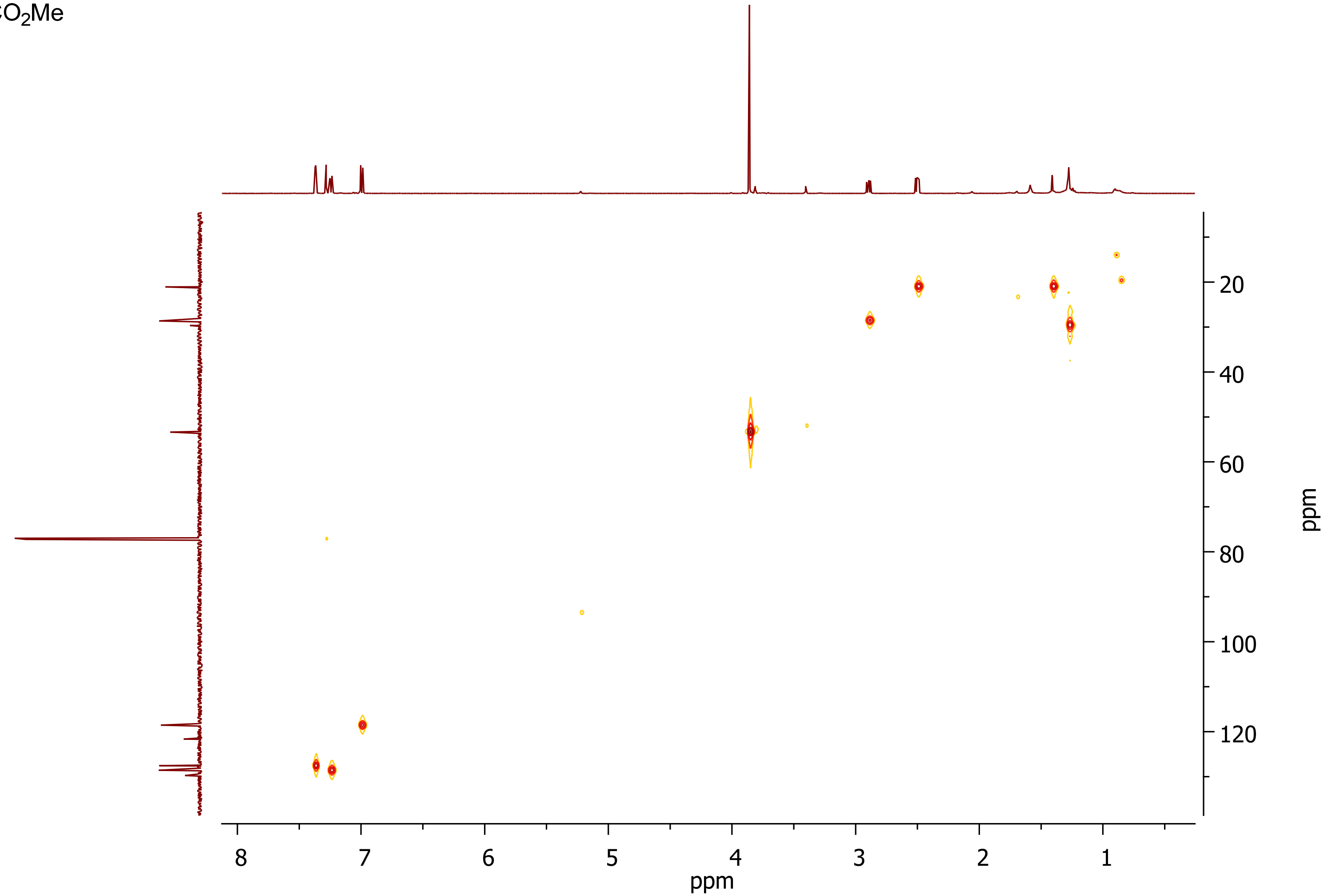

Methyl (1a*RS*,7b*RS*)-6-bromo-2-oxo-1,7b-dihydrocyclopropa[*c*]chromene-1a(2*H*)-carboxylate (2c)

^{1}H NMR ($CDCl_3$, 500 MHz)

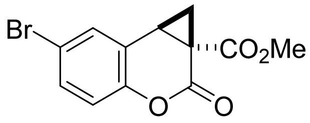

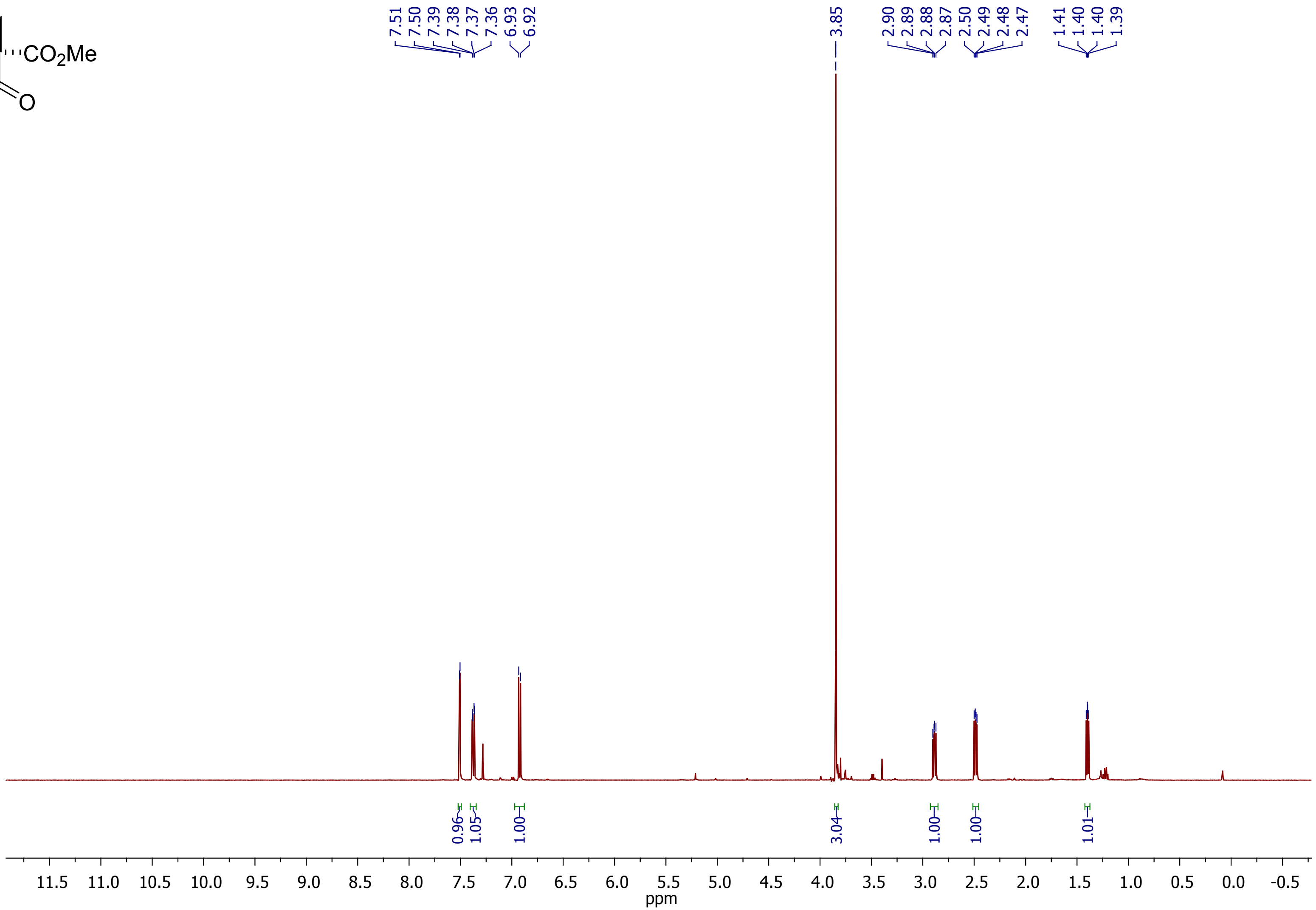

Methyl (1a*RS*,7b*RS*)-6-bromo-2-oxo-1,7b-dihydrocyclopropa[*c*]chromene-1a(2*H*)-carboxylate (2c)

^{13}C NMR ($CDCl_3$, 125 MHz)

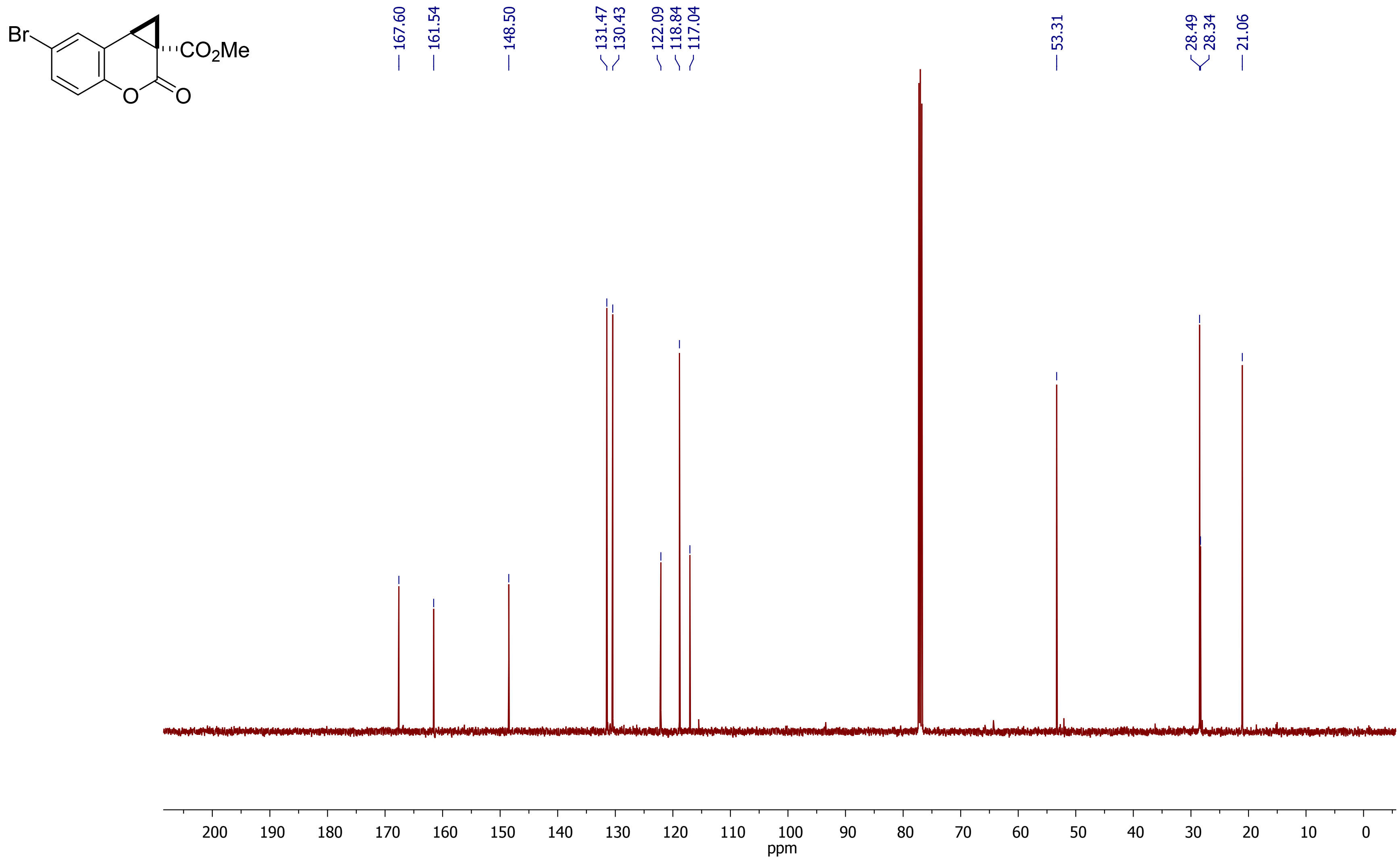

Methyl (1a*RS*,7b*RS*)-6-bromo-2-oxo-1,7b-dihydrocyclopropa[*c*]chromene-1a(2*H*)-carboxylate (2c)

HSQC ^{1}H-^{13}C ($CDCl_3$)

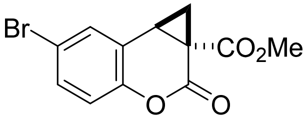

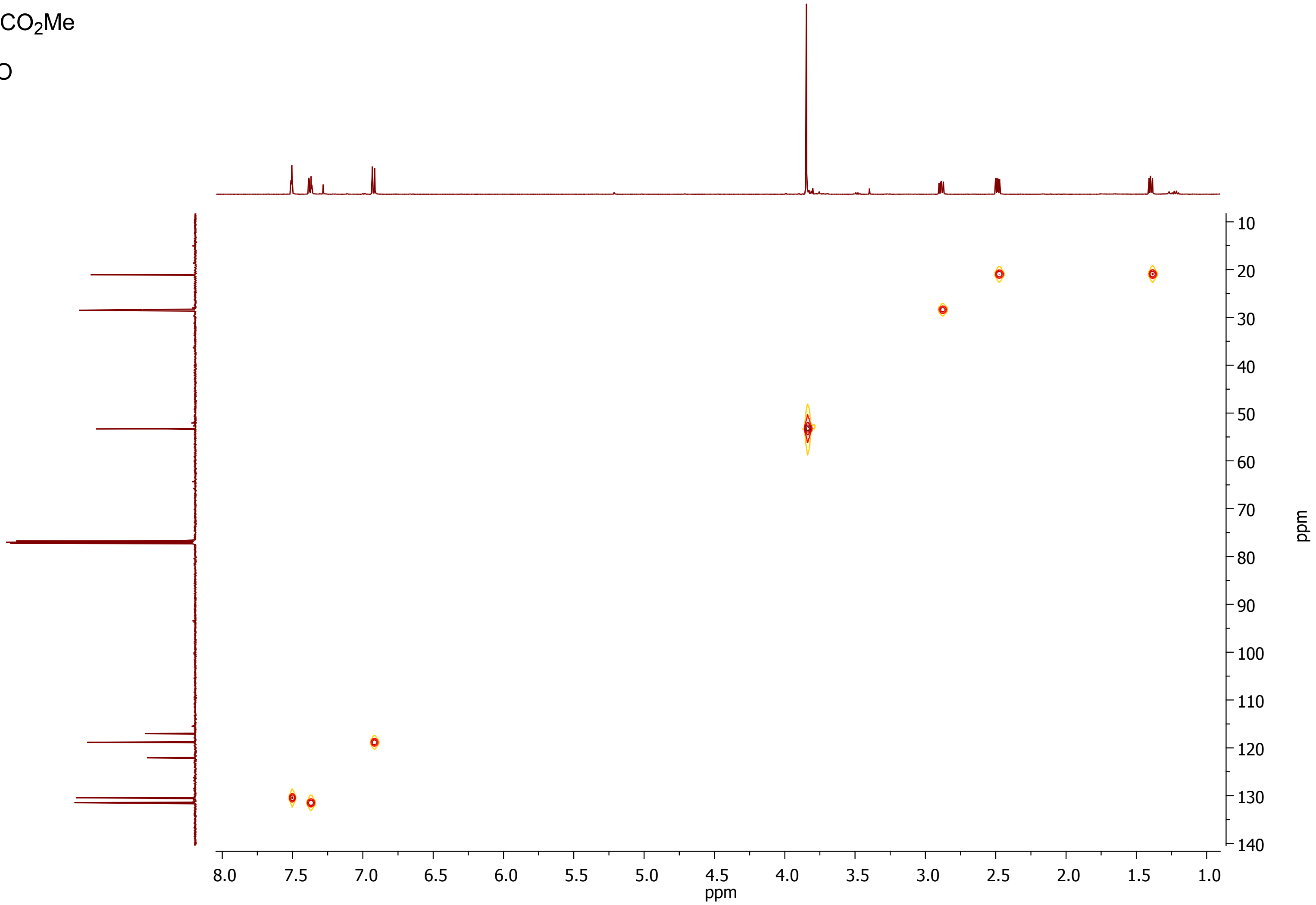

Methyl 6-bromo-2-oxo-1,7b-dihydrocyclopropa[*c*]chromene-1a(2*H*)-carboxylate (2c)

HMBC ^{1}H-^{13}C ($CDCl_3$)

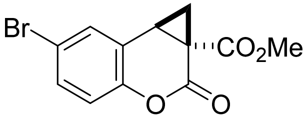

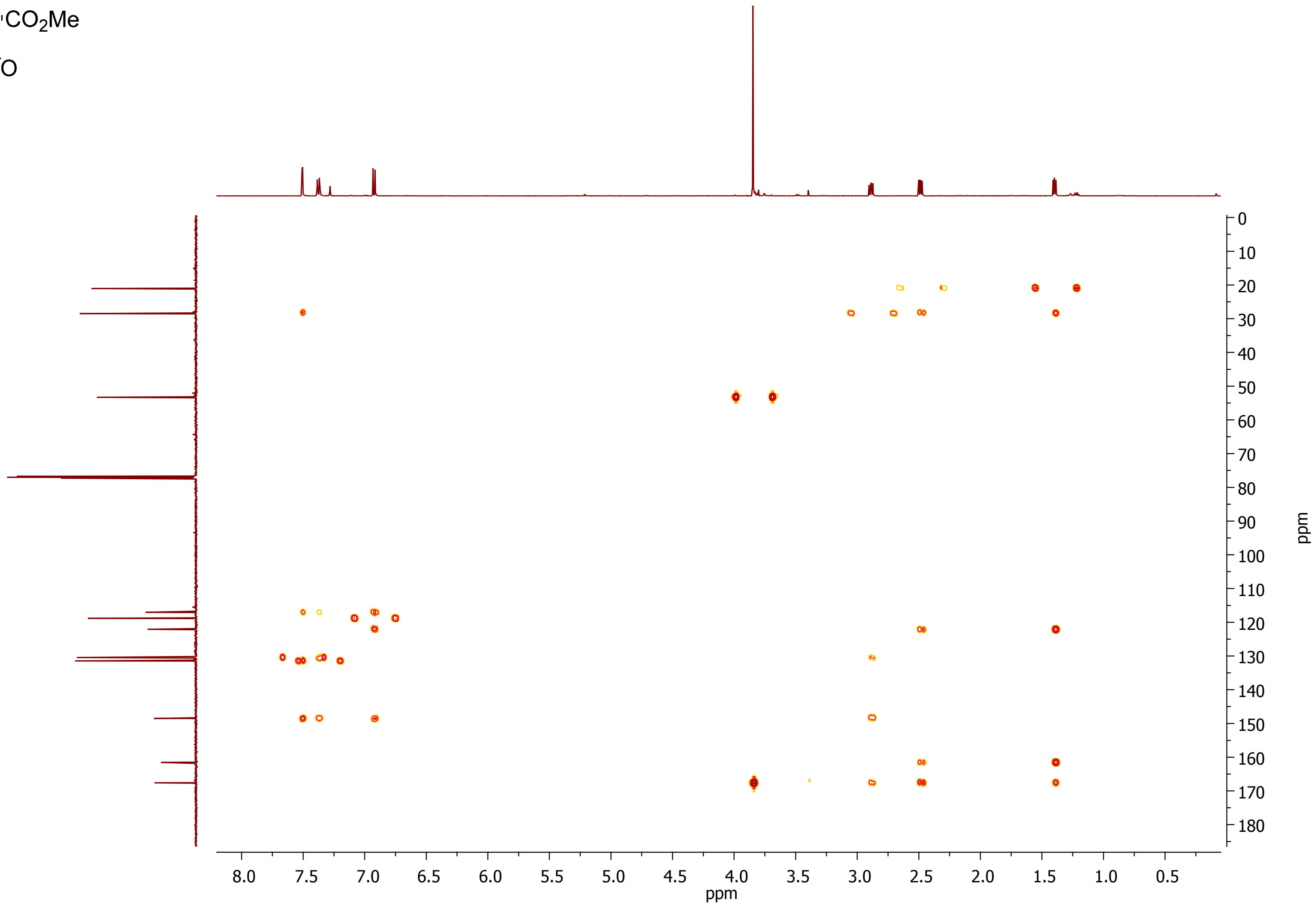

Methyl (1a*RS*,7b*RS*)-6-fluoro-2-oxo-1,7b-dihydrocyclopropa[c]chromene-1a(2*H*)-carboxylate (2d)

^{1}H NMR ($CDCl_3$, 400 MHz)

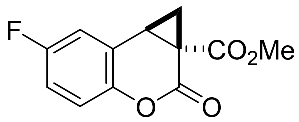

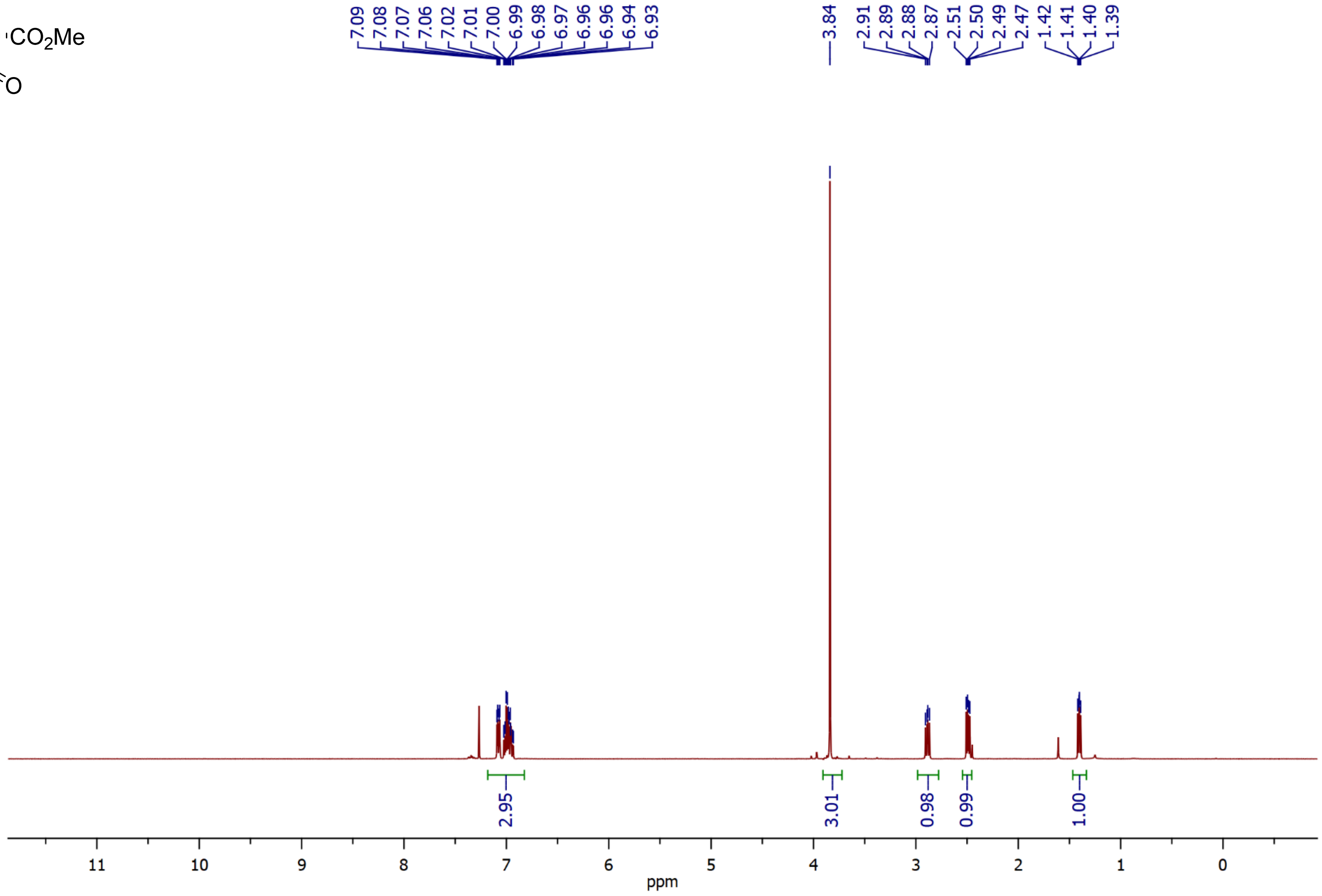

Methyl (1a*RS*,7b*RS*)-6-fluoro-2-oxo-1,7b-dihydrocyclopropa[c]chromene-1a(2*H*)-carboxylate (2d)

^{13}C NMR ($CDCl_3$, 100 MHz)

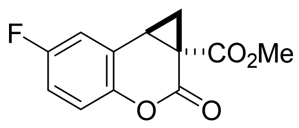

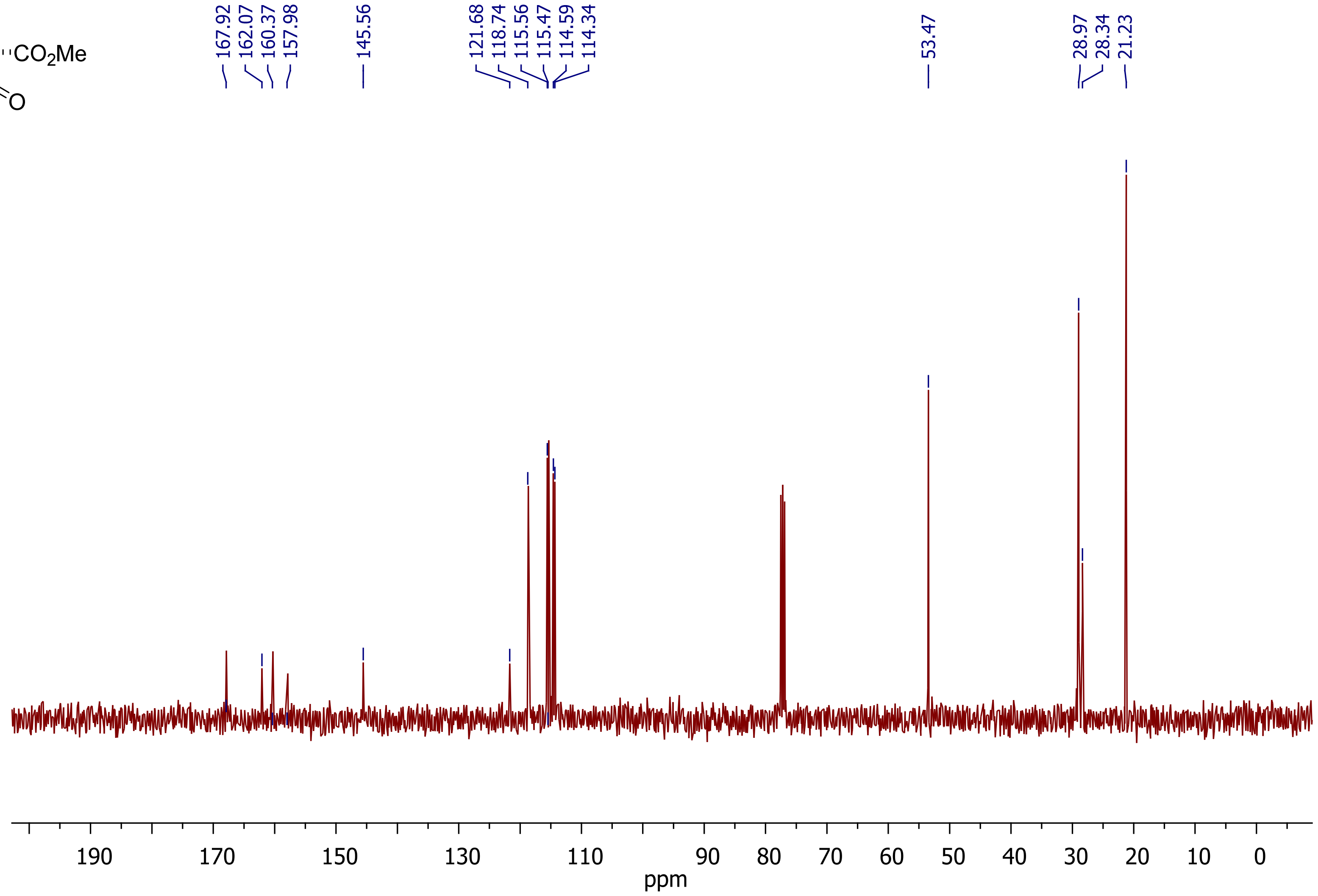

Methyl (1a*RS*,7b*RS*)-6-fluoro-2-oxo-1,7b-dihydrocyclopropa[c]chromene-1a(2*H*)-carboxylate (2d)

HSQC ^{1}H-^{13}C ($CDCl_3$)

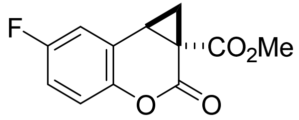

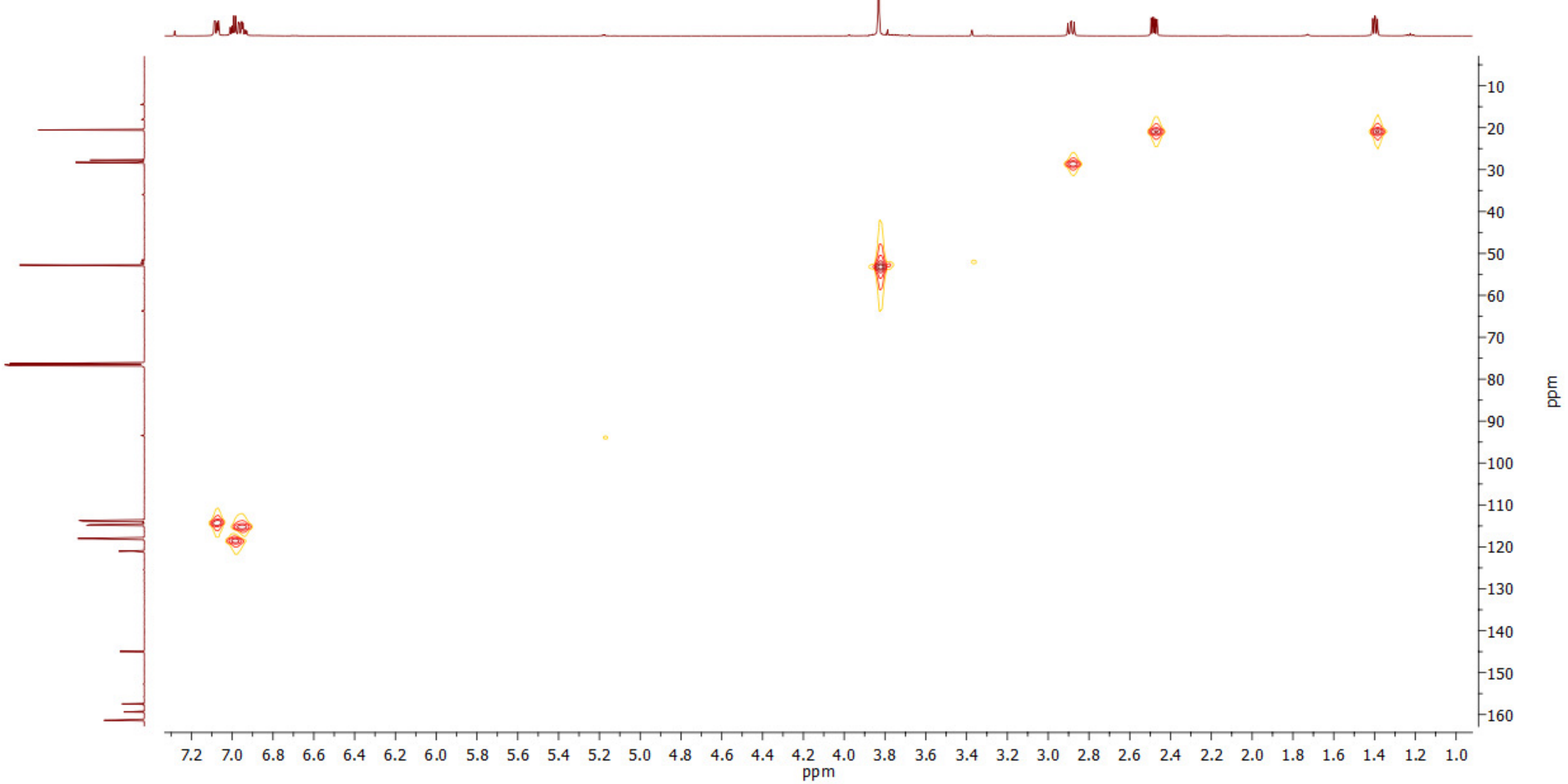

Methyl (1a*RS*,7b*RS*)-6-fluoro-2-oxo-1,7b-dihydrocyclopropa[c]chromene-1a(2*H*)-carboxylate (2d)

HMBC ^{1}H-^{13}C ($CDCl_3$)

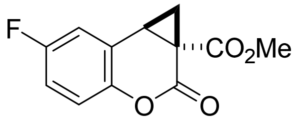

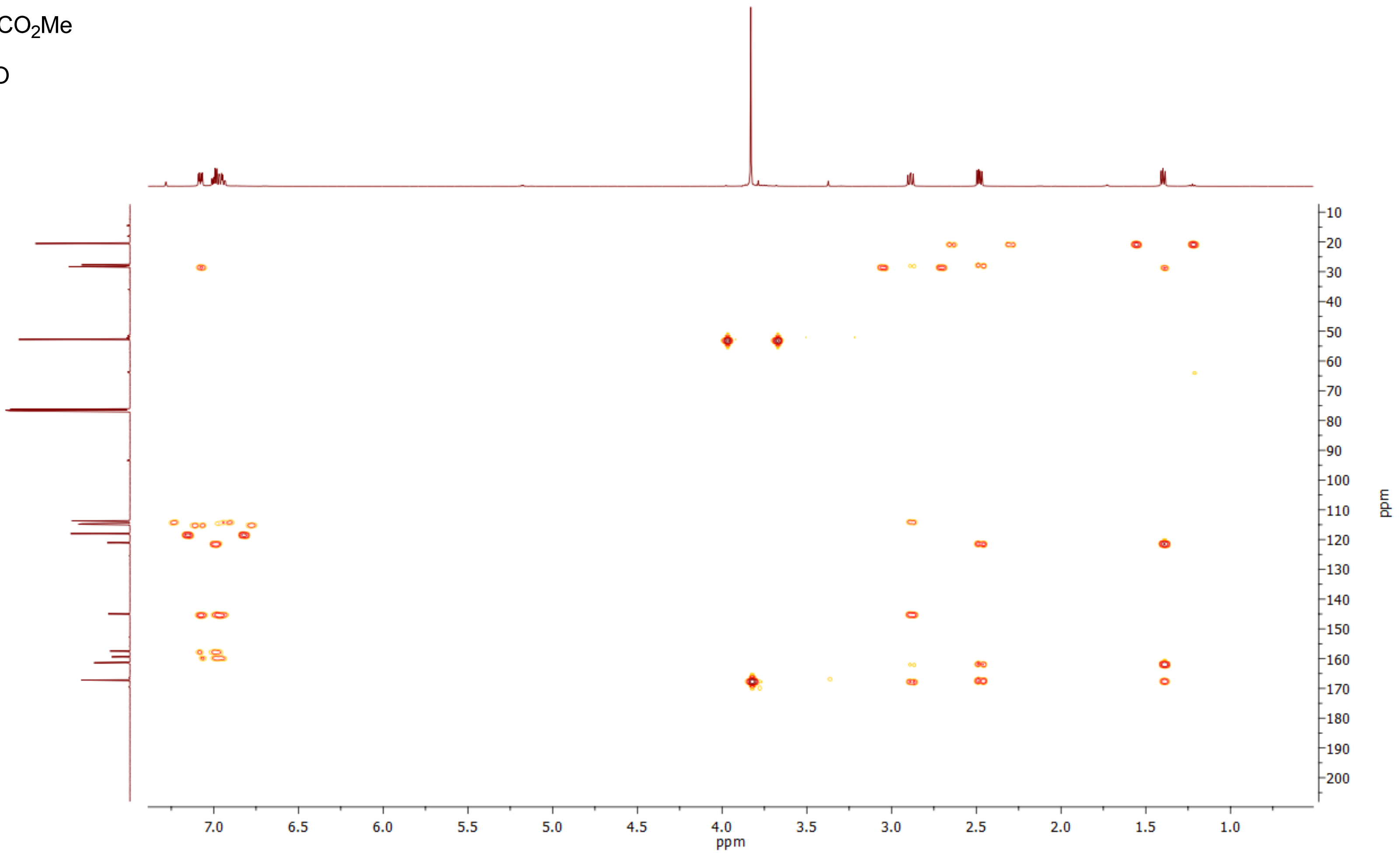

Methyl (1a*RS*,7b*RS*)-6-bromo-2-oxo-1,7b-dihydrocyclopropa[*c*]chromene-1a(2*H*)-carboxylate (2e)

^{1}H NMR ($CDCl_3$, 500 MHz)

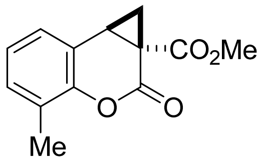

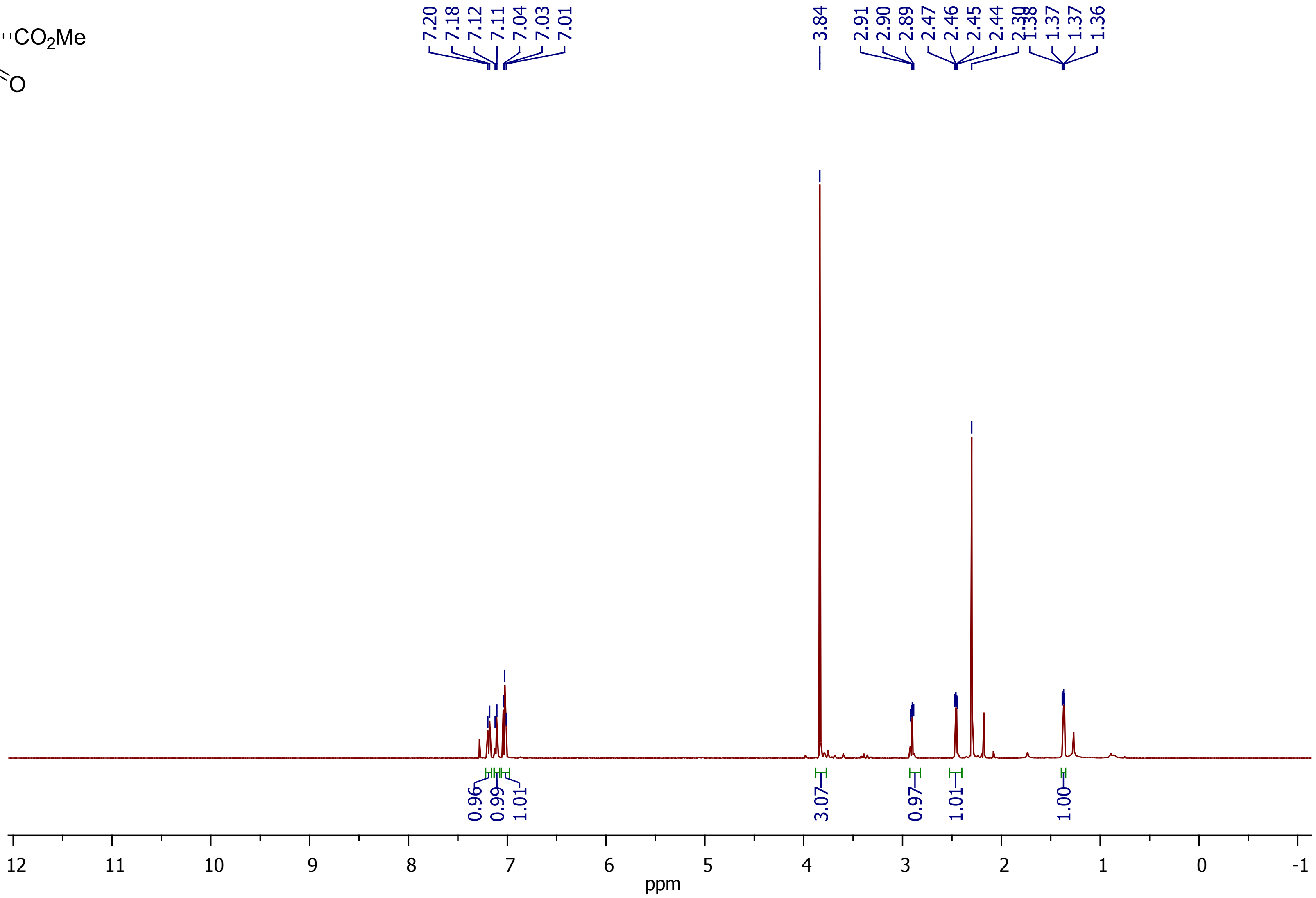

Methyl (1a*RS*,7b*RS*)-6-bromo-2-oxo-1,7b-dihydrocyclopropa[*c*]chromene-1a(2*H*)-carboxylate (2e)

^{13}C NMR ($CDCl_3$, 125 MHz)

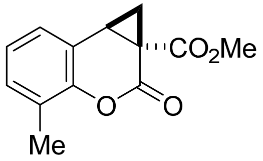

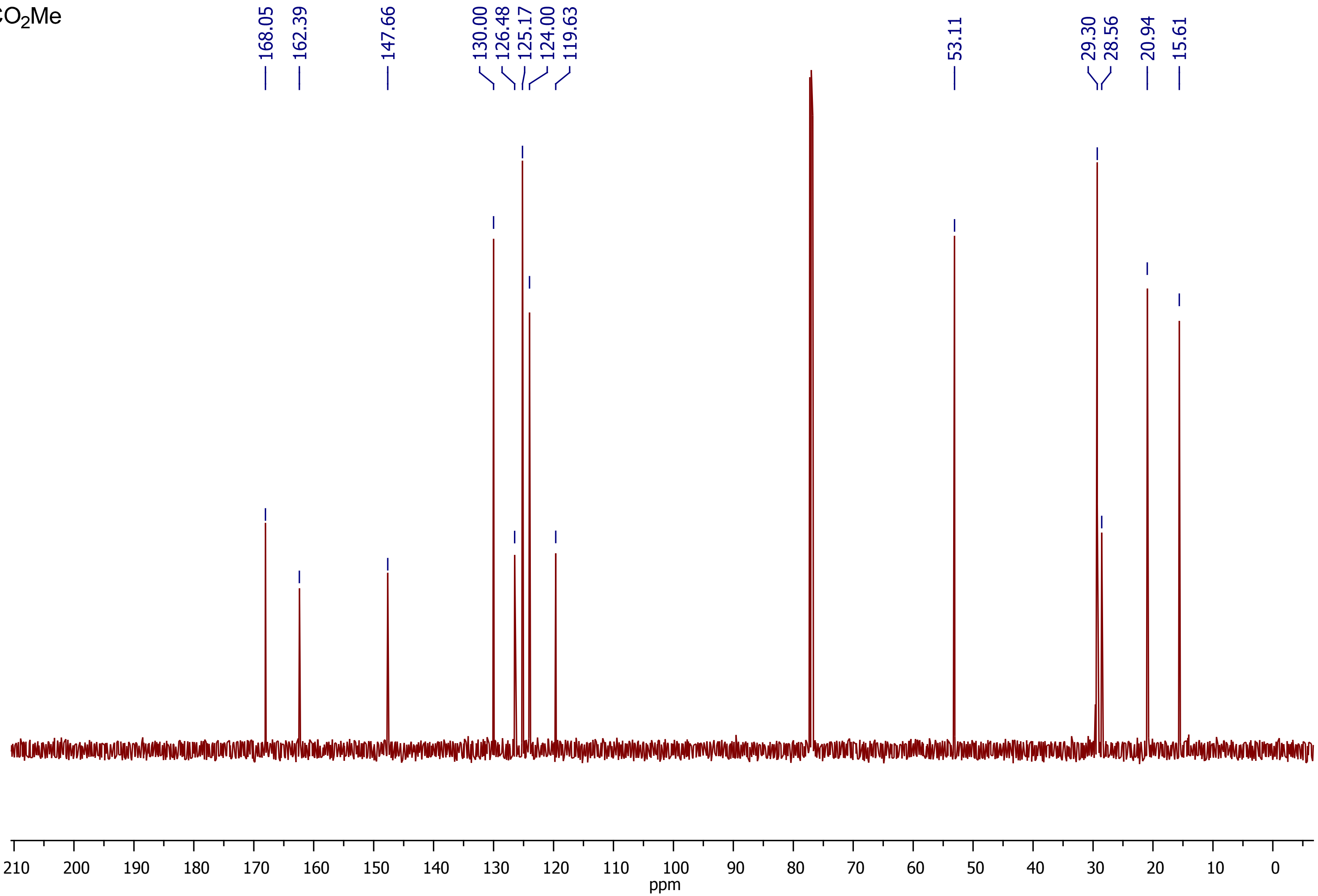

Methyl (1a*RS*,7b*RS*)-6-bromo-2-oxo-1,7b-dihydrocyclopropa[*c*]chromene-1a(2*H*)-carboxylate (2e)

HSQC ^{1}H-^{13}C ($CDCl_3$)

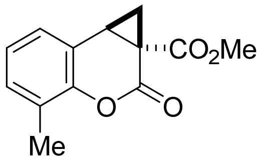

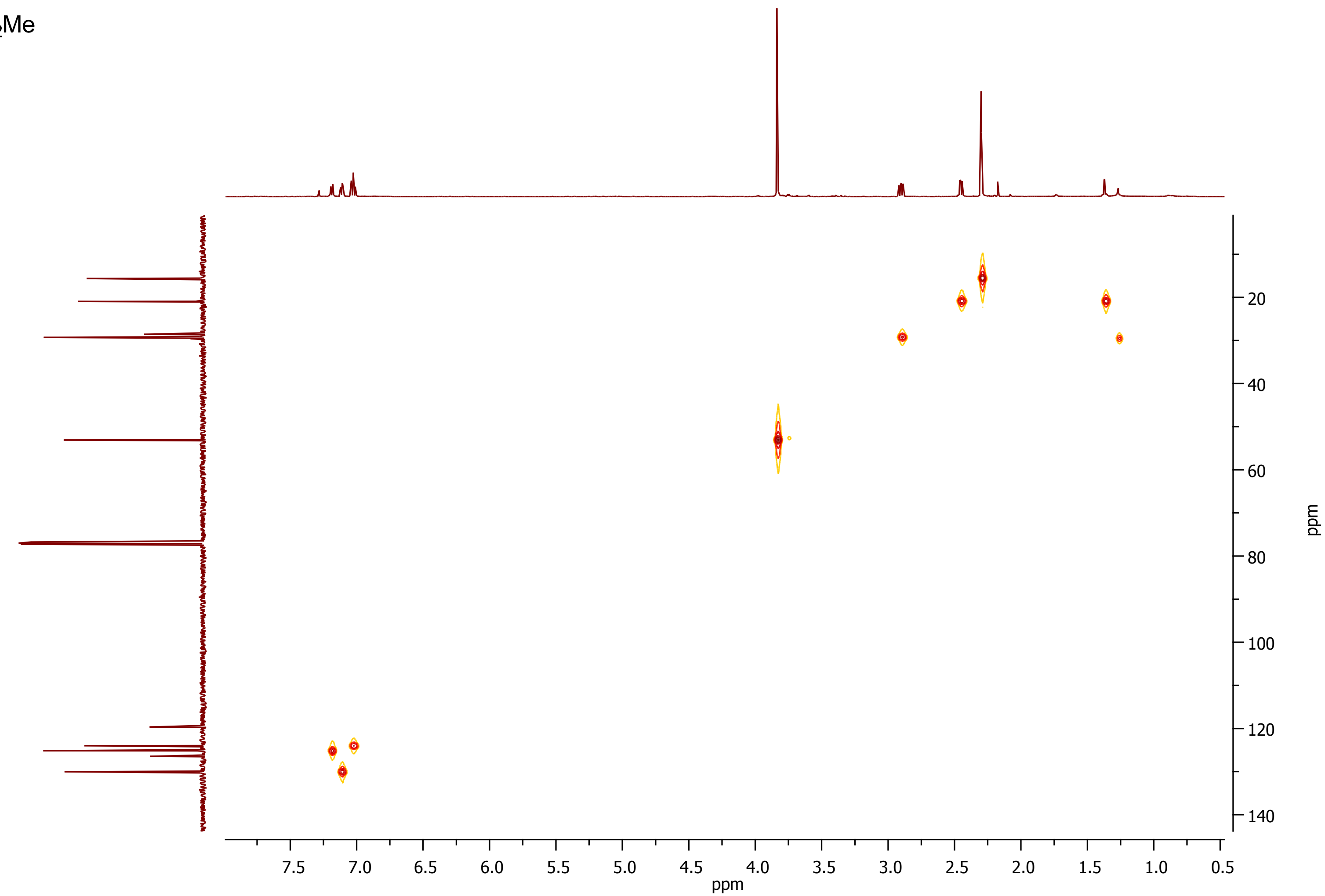

Methyl (1a*RS*,7b*RS*)-6-bromo-2-oxo-1,7b-dihydrocyclopropa[*c*]chromene-1a(2*H*)-carboxylate (2e)

HMBC ^{1}H-^{13}C ($CDCl_3$)

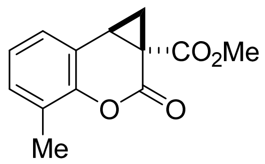

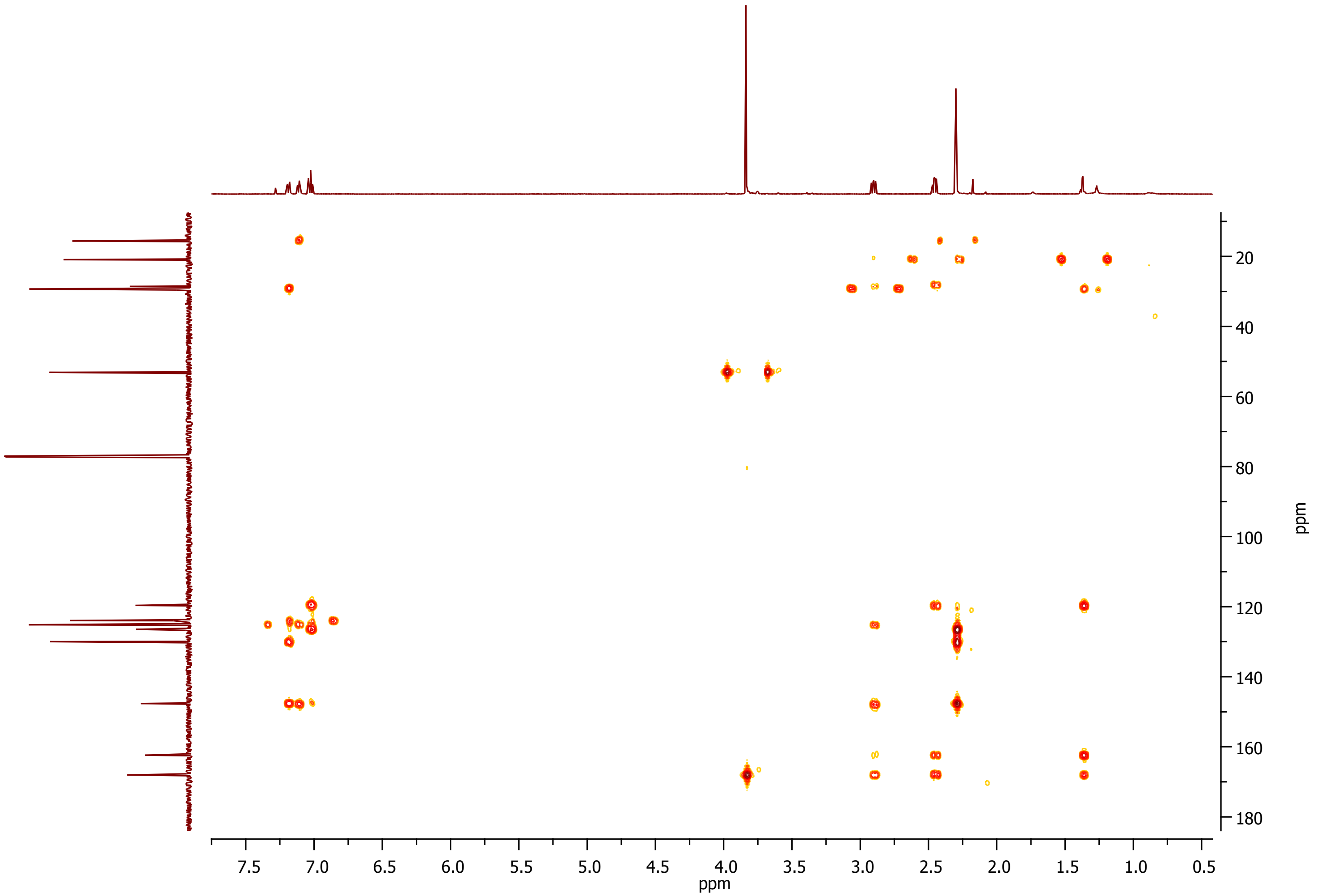

Methyl (1a*RS*,7b*RS*)-4,6-dibromo-2-oxo-1,7b-dihydrocyclopropa[*c*]chromene-1a(2*H*)-carboxylate (2f)

^{1}H NMR ($CDCl_3$, 500 MHz)

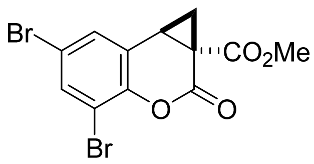

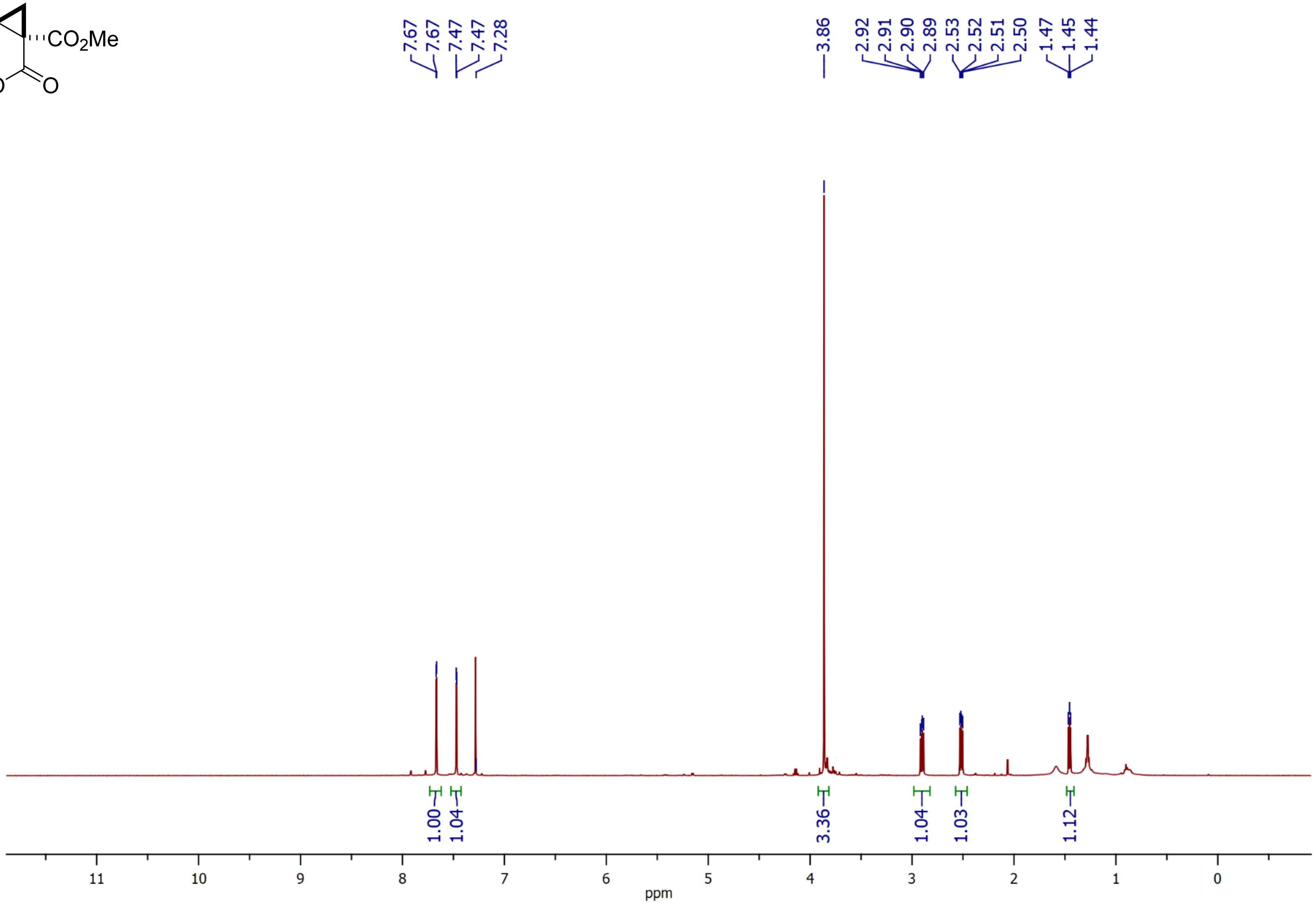

Methyl (1a*RS*,7b*RS*)-4,6-dibromo-2-oxo-1,7b-dihydrocyclopropa[*c*]chromene-1a(2*H*)-carboxylate (2f)

^{13}C NMR ($CDCl_3$, 125 MHz)

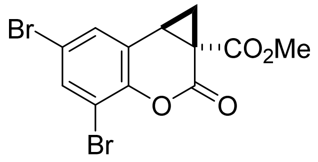

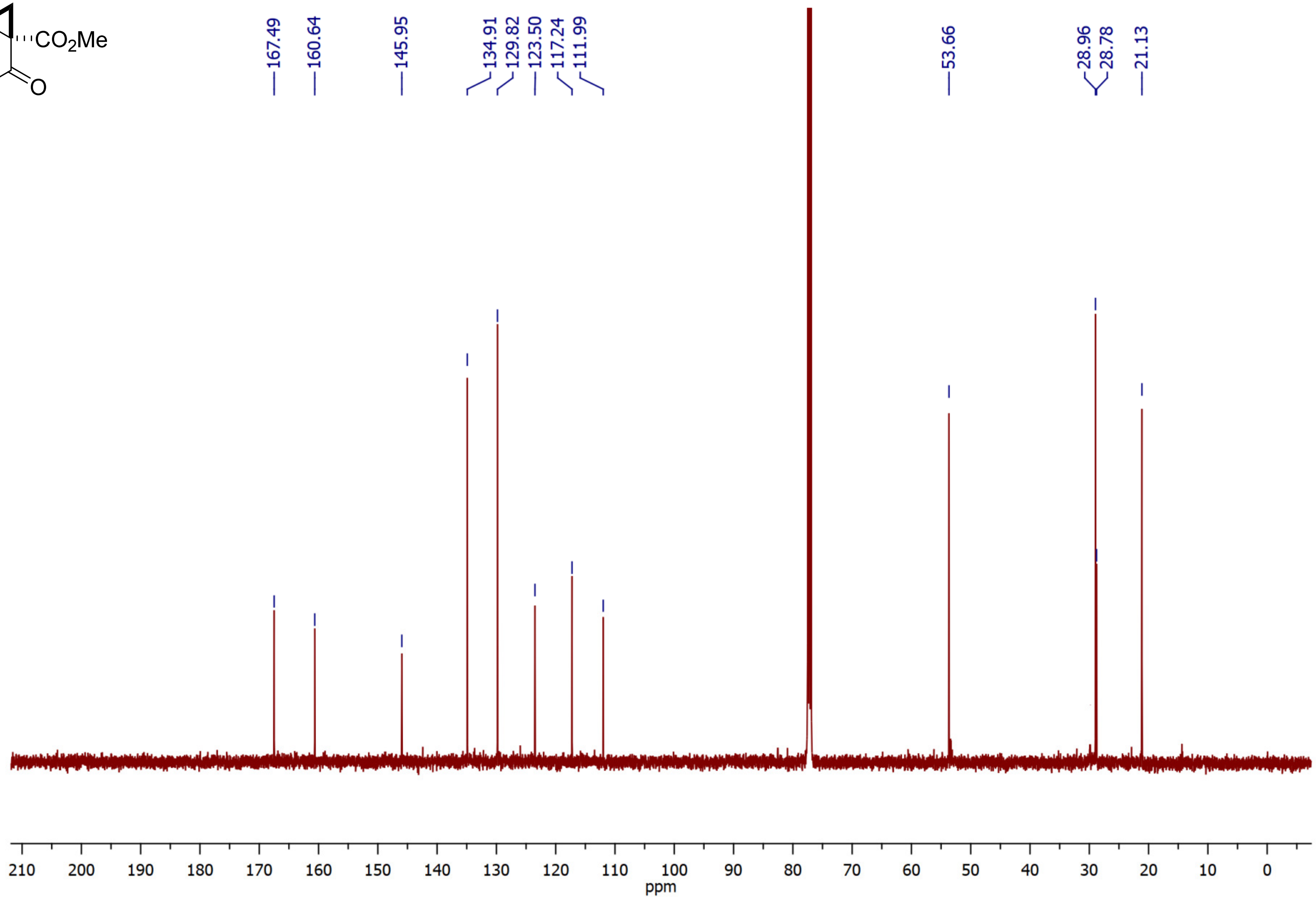

Methyl (1a*RS*,7b*RS*)-6-nitro-2-oxo-1,7b-dihydrocyclopropa[*c*]chromene-1a(2*H*)-carboxylate (2h)

^{1}H NMR ($CDCl_3$, 500 MHz)

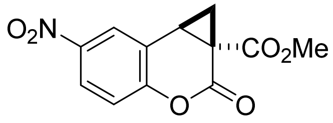

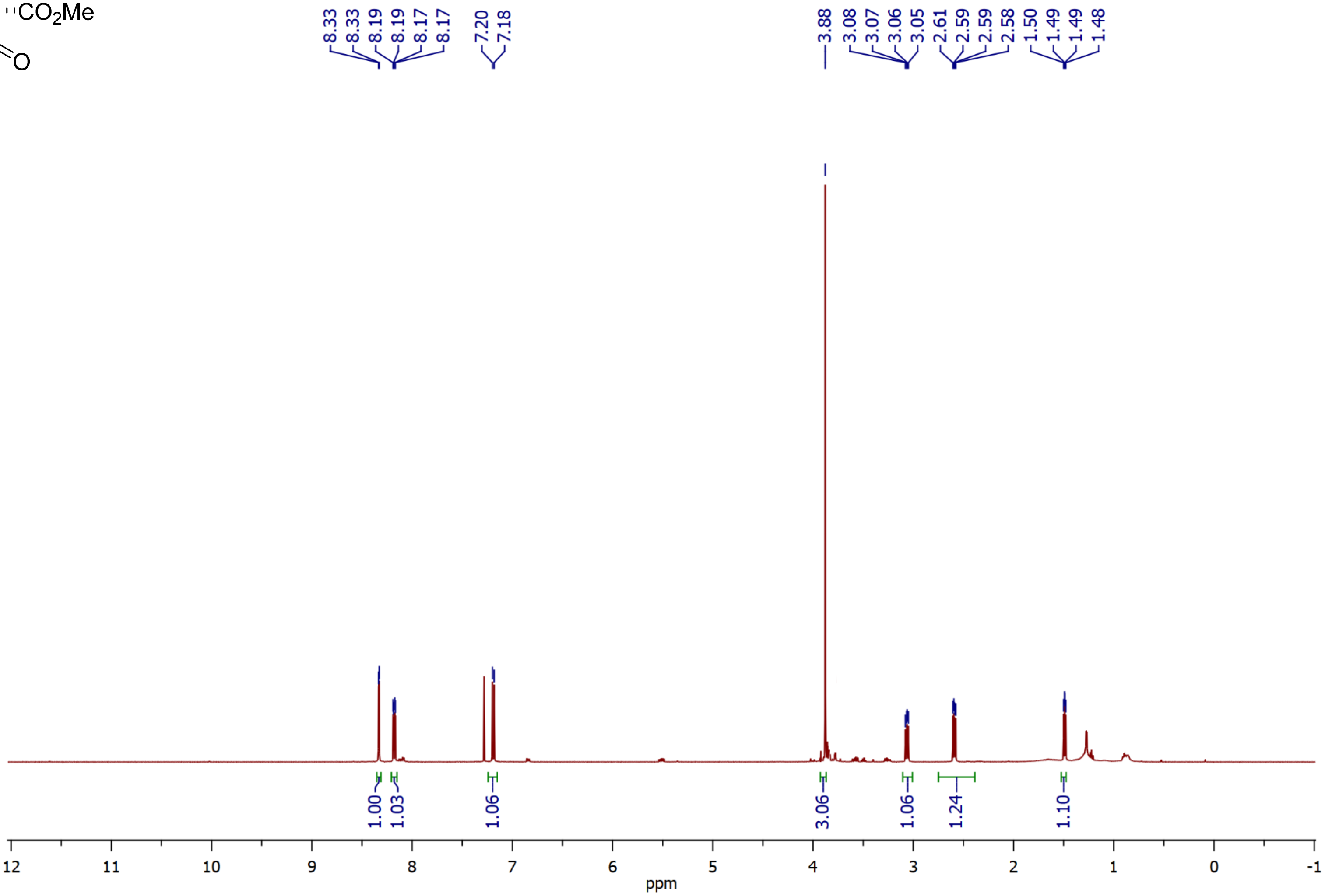

Methyl (1a*RS*,7b*RS*)-6-nitro-2-oxo-1,7b-dihydrocyclopropa[*c*]chromene-1a(2*H*)-carboxylate (2h)

^{13}C NMR ($CDCl_3$, 125 MHz)

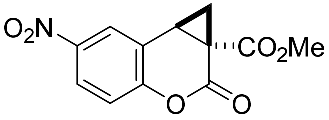

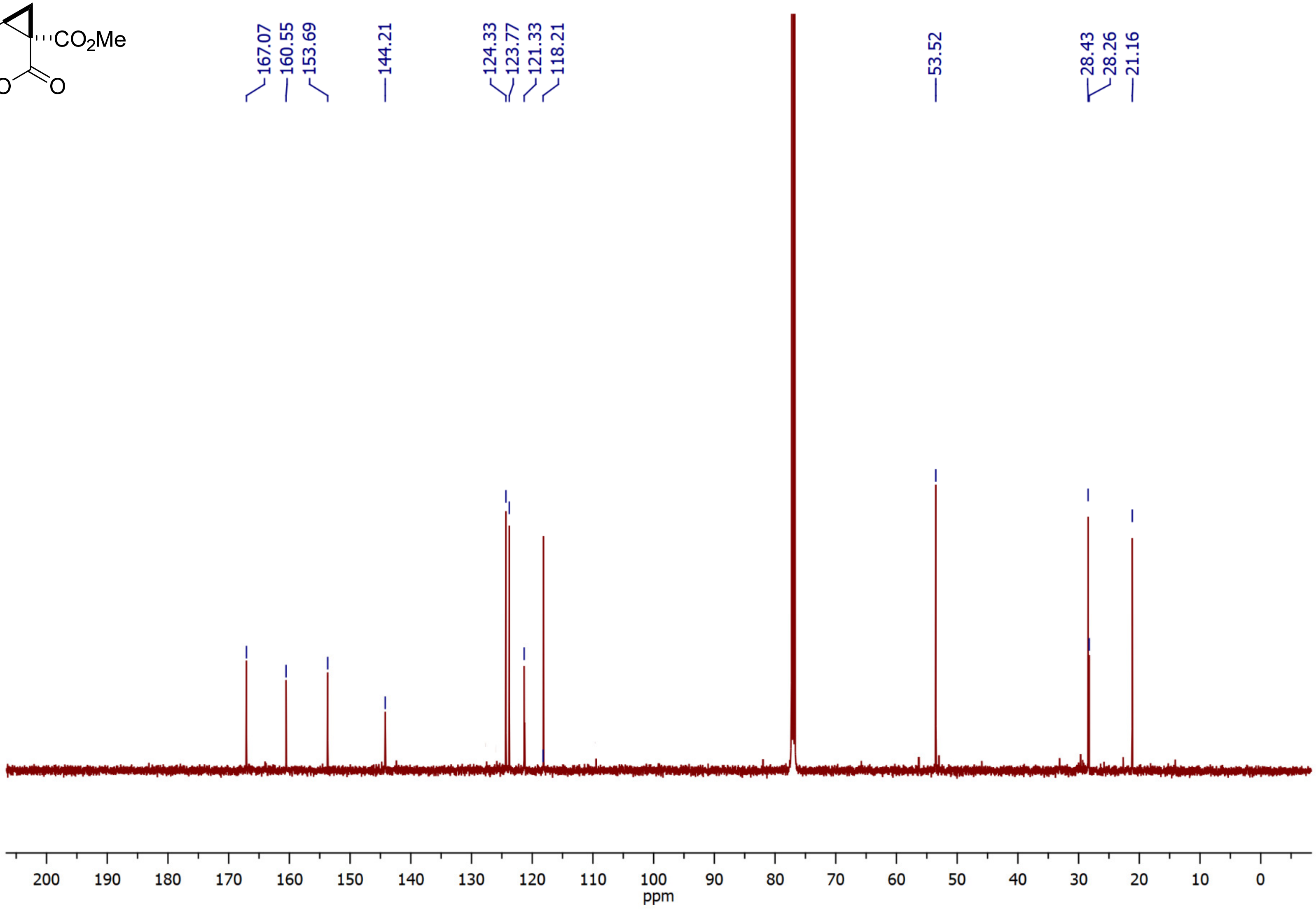

Dimethyl 2-[2-(acetoxymethyl)phenyl]cyclopropane-1,1-dicarboxylate (5)

^{1}H NMR ($CDCl_3$, 400 MHz)

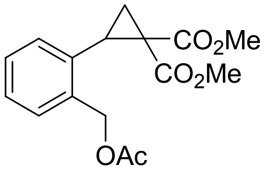

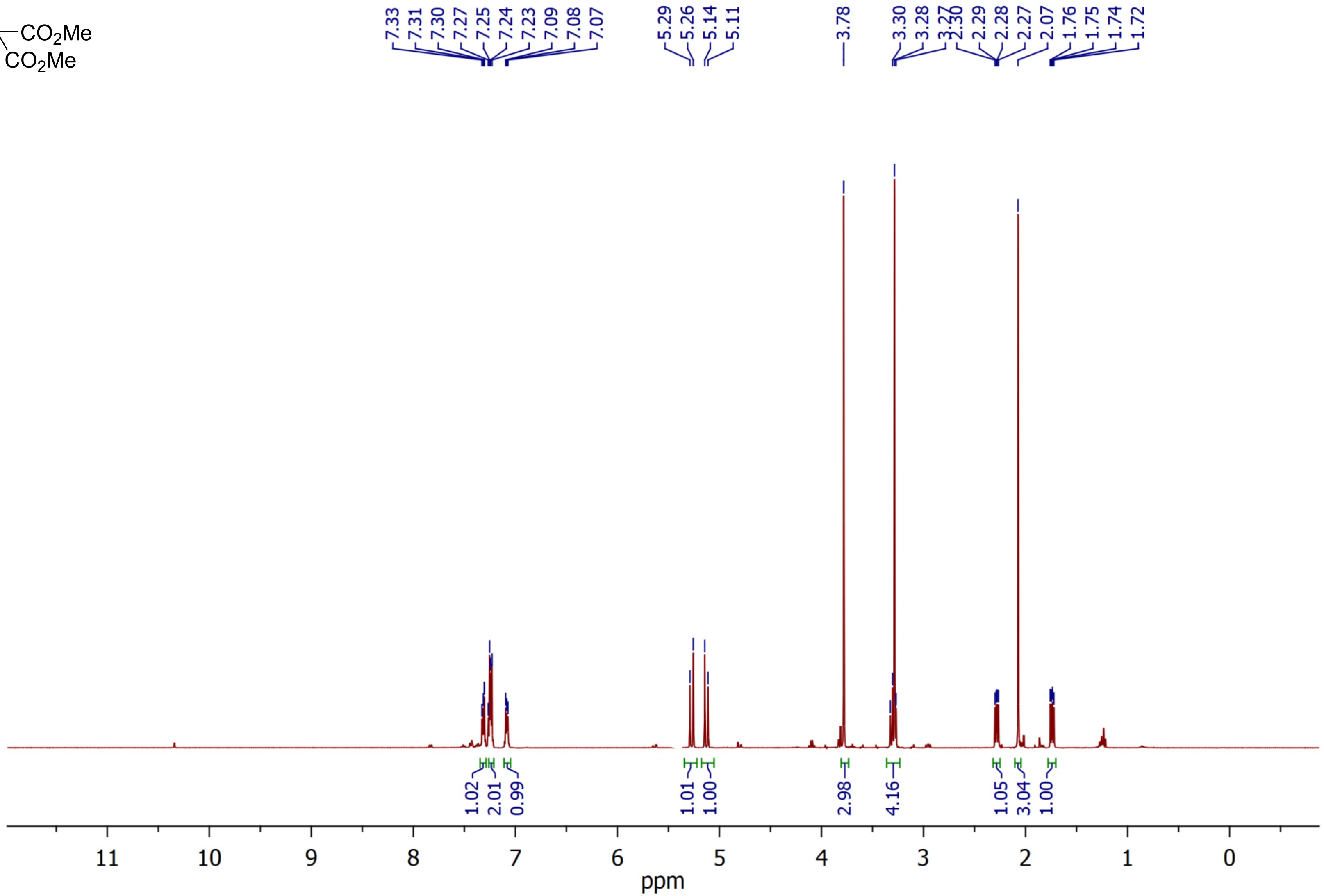

Dimethyl 2-[2-(acetoxymethyl)phenyl]cyclopropane-1,1-dicarboxylate (5)

^{13}C NMR ($CDCl_3$, 100 MHz)

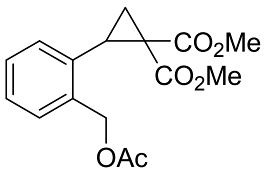

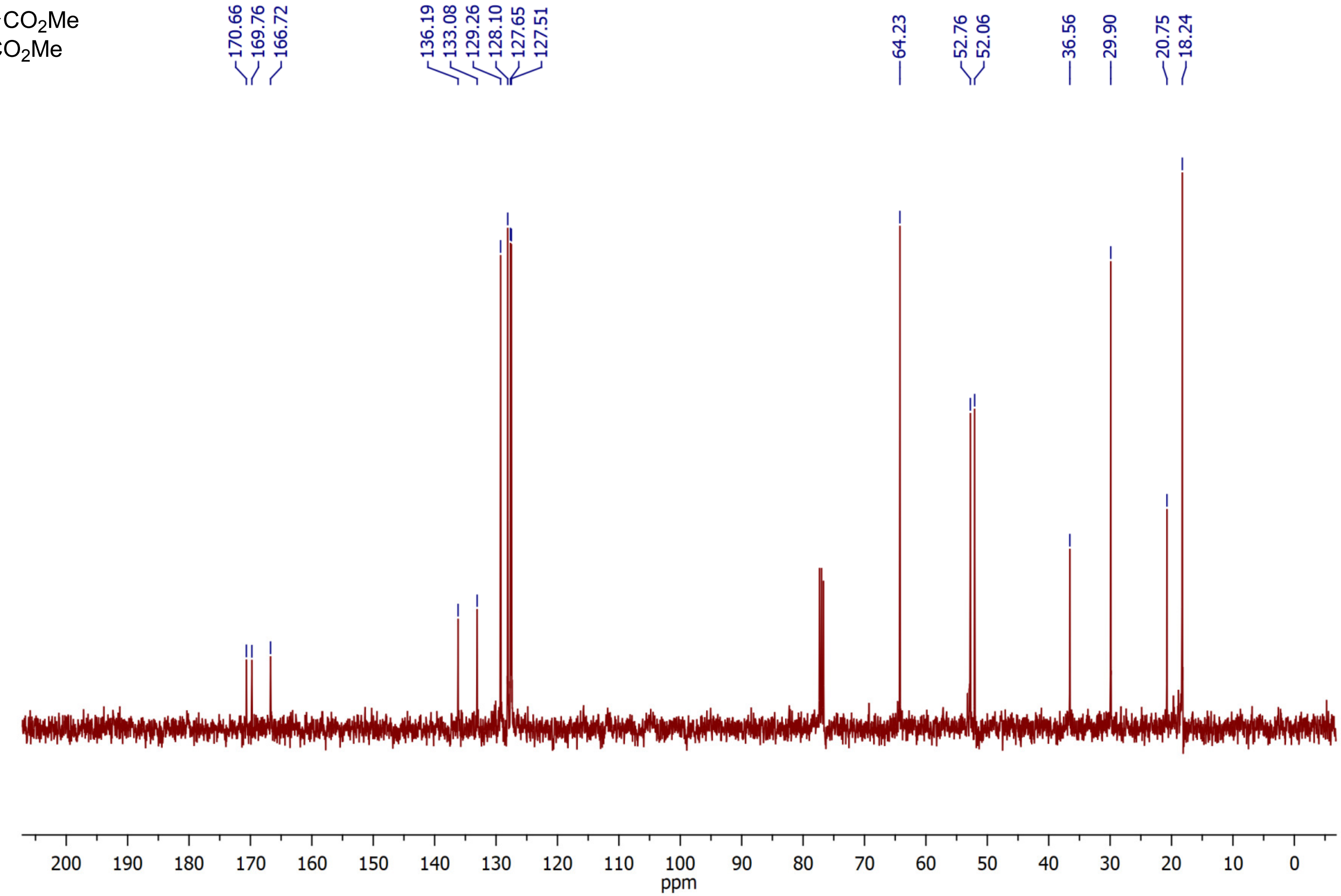

Dimethyl 2-(3-formyl-1-methyl-1*H*-indol-4-yl)cyclopropane-1,1-dicarboxylate (7)

^{1}H NMR ($CDCl_3$, 500 MHz)

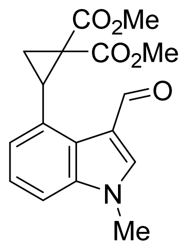

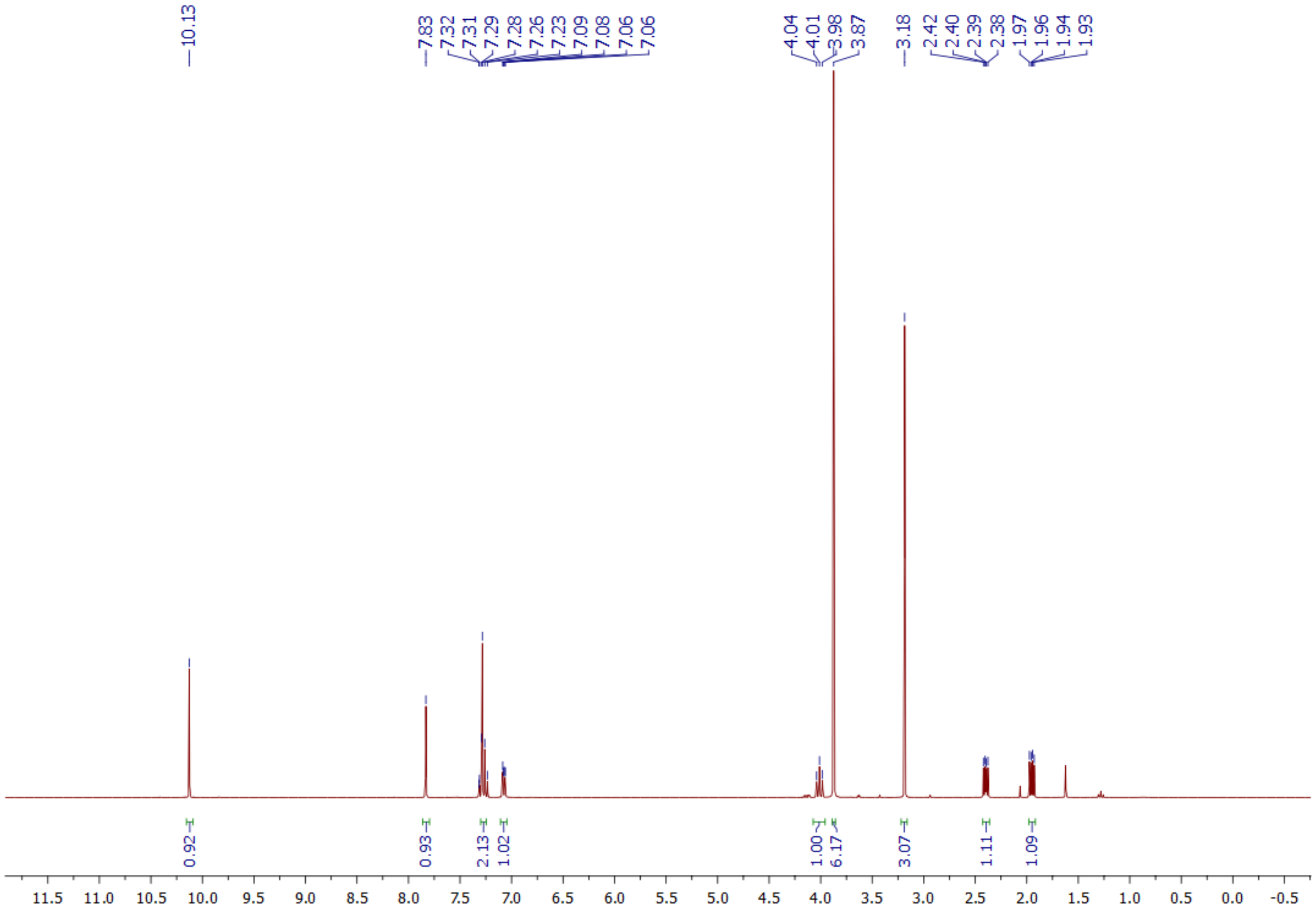

Dimethyl 2-(3-formyl-1-methyl-1*H*-indol-4-yl)cyclopropane-1,1-dicarboxylate (7)

^{13}C NMR ($CDCl_3$, 125 MHz)

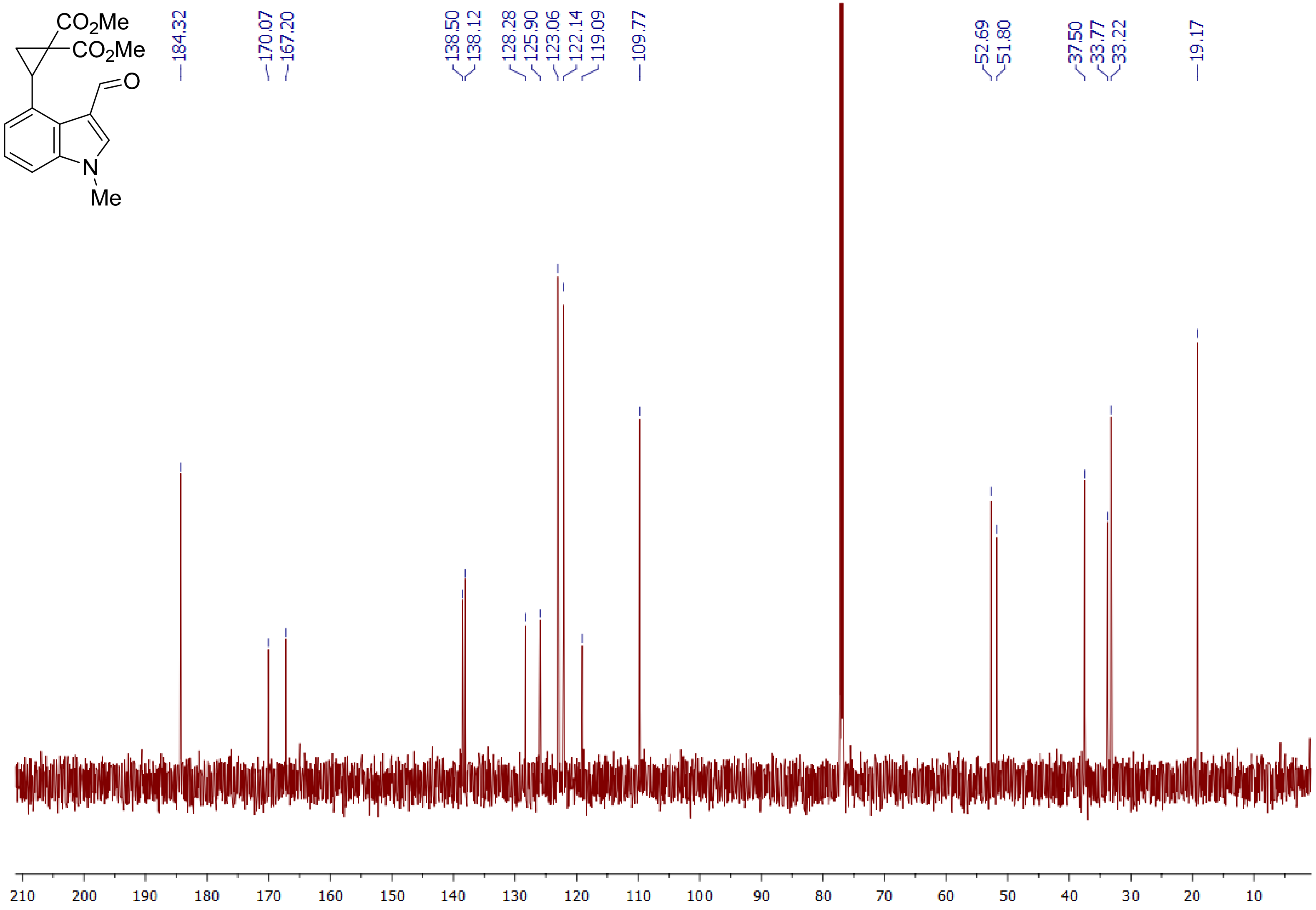

Dimethyl 2-(3-hydroxymethyl-1-methyl-1*H*-indol-4-yl)cyclopropane-1,1-dicarboxylate (8)

^{1}H NMR ($CDCl_3$, 500 MHz)

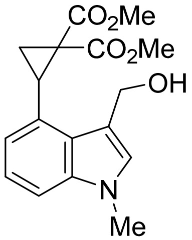

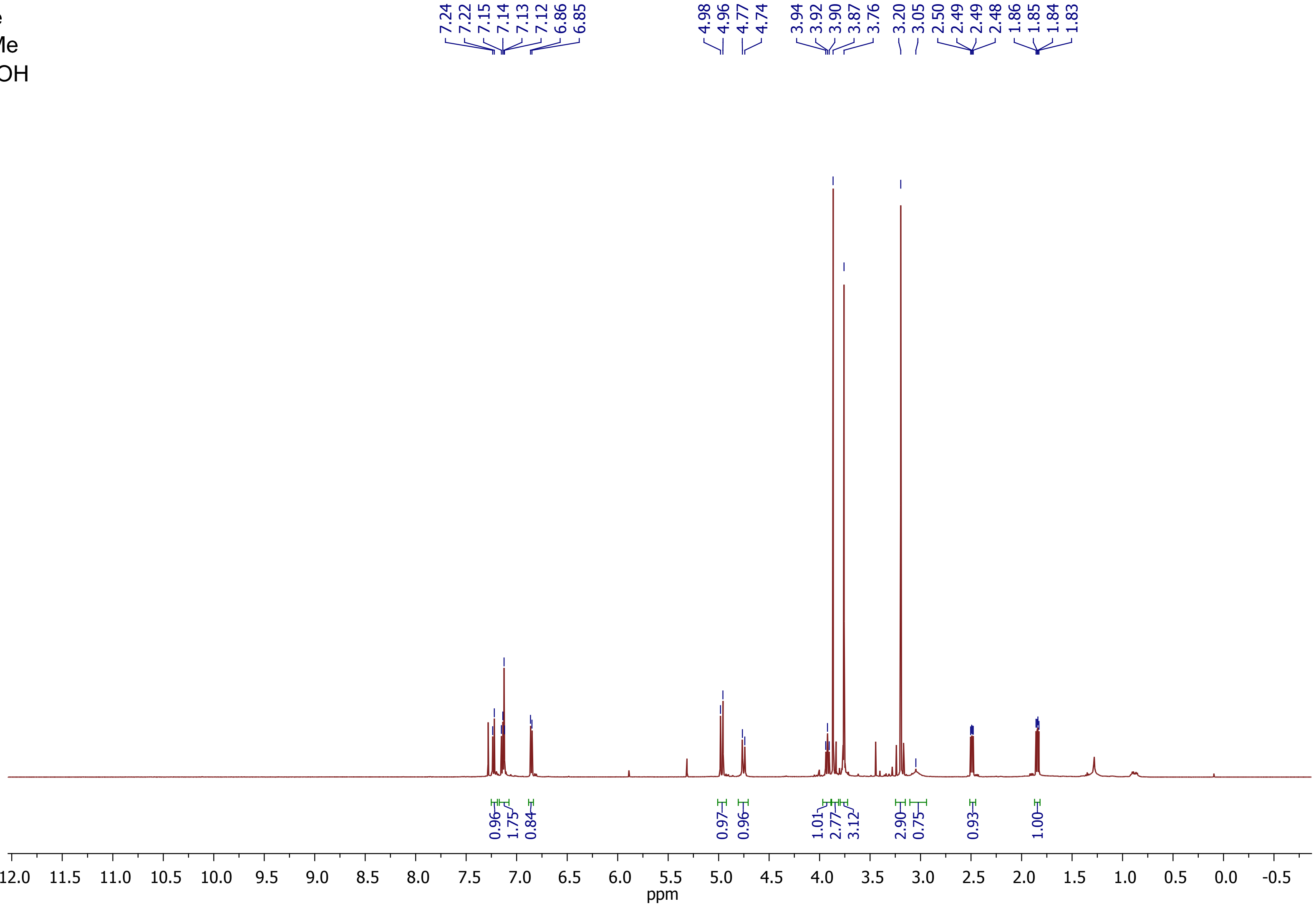

Dimethyl 2-(3-hydroxymethyl-1-methyl-1*H*-indol-4-yl)cyclopropane-1,1-dicarboxylate (8)

^{13}C NMR ($CDCl_3$, 125 MHz)

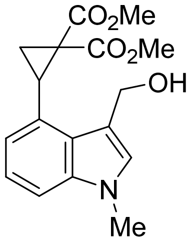

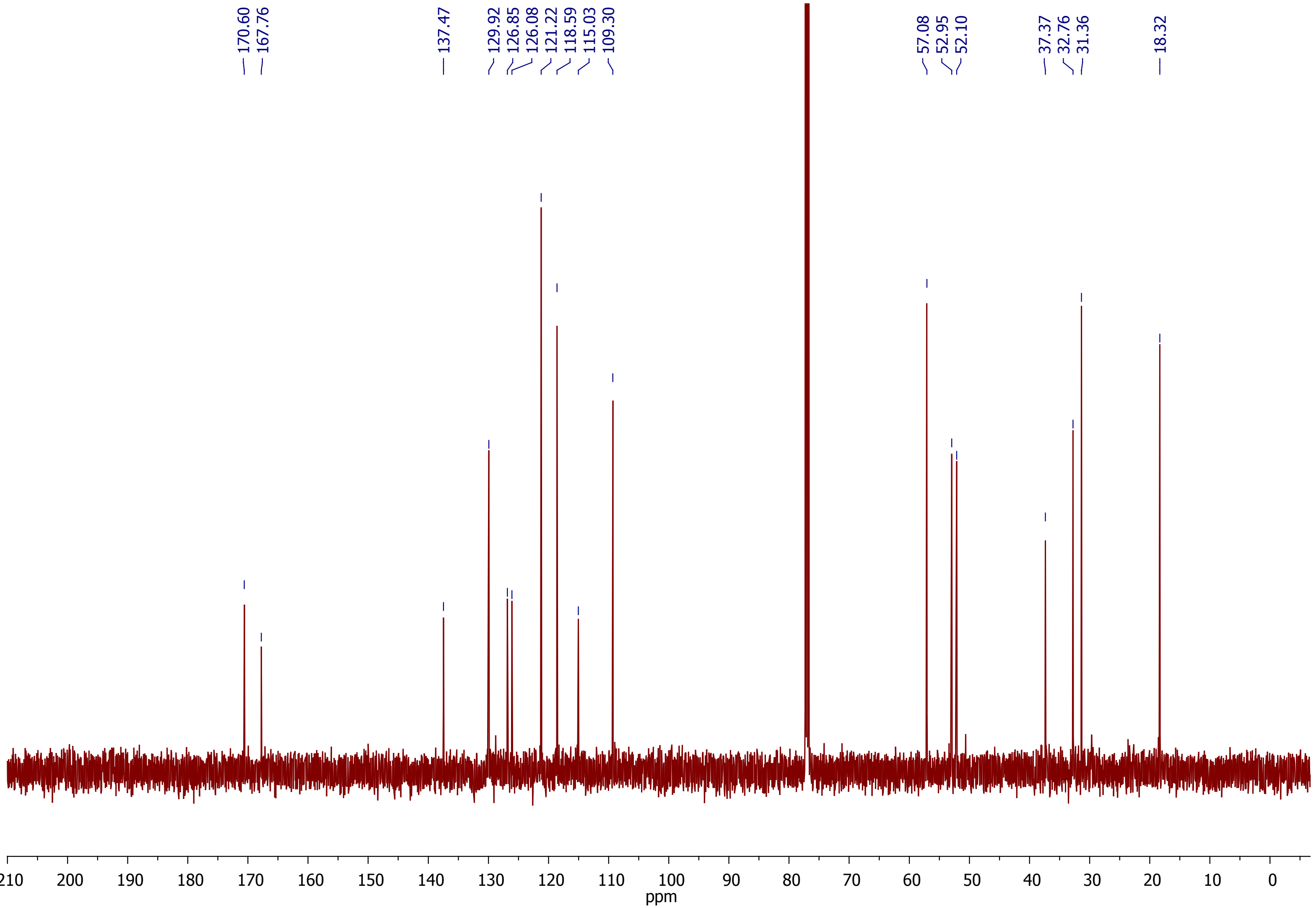

Dimethyl 2-[3-({4-[2,2-bis(methoxycarbonyl)cyclopropyl]-1-methyl-1*H*-indol-3-yl}methyl)-1-methyl-1*H*-indol-4-yl]cyclopropane-1,1-dicarboxylate (9)

^{1}H NMR ($CDCl_3$, 500 MHz)

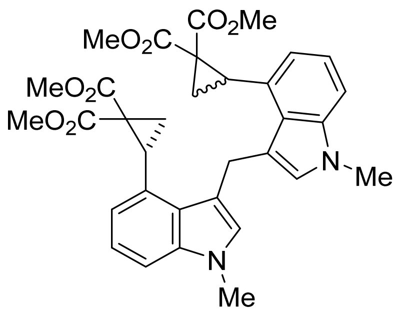

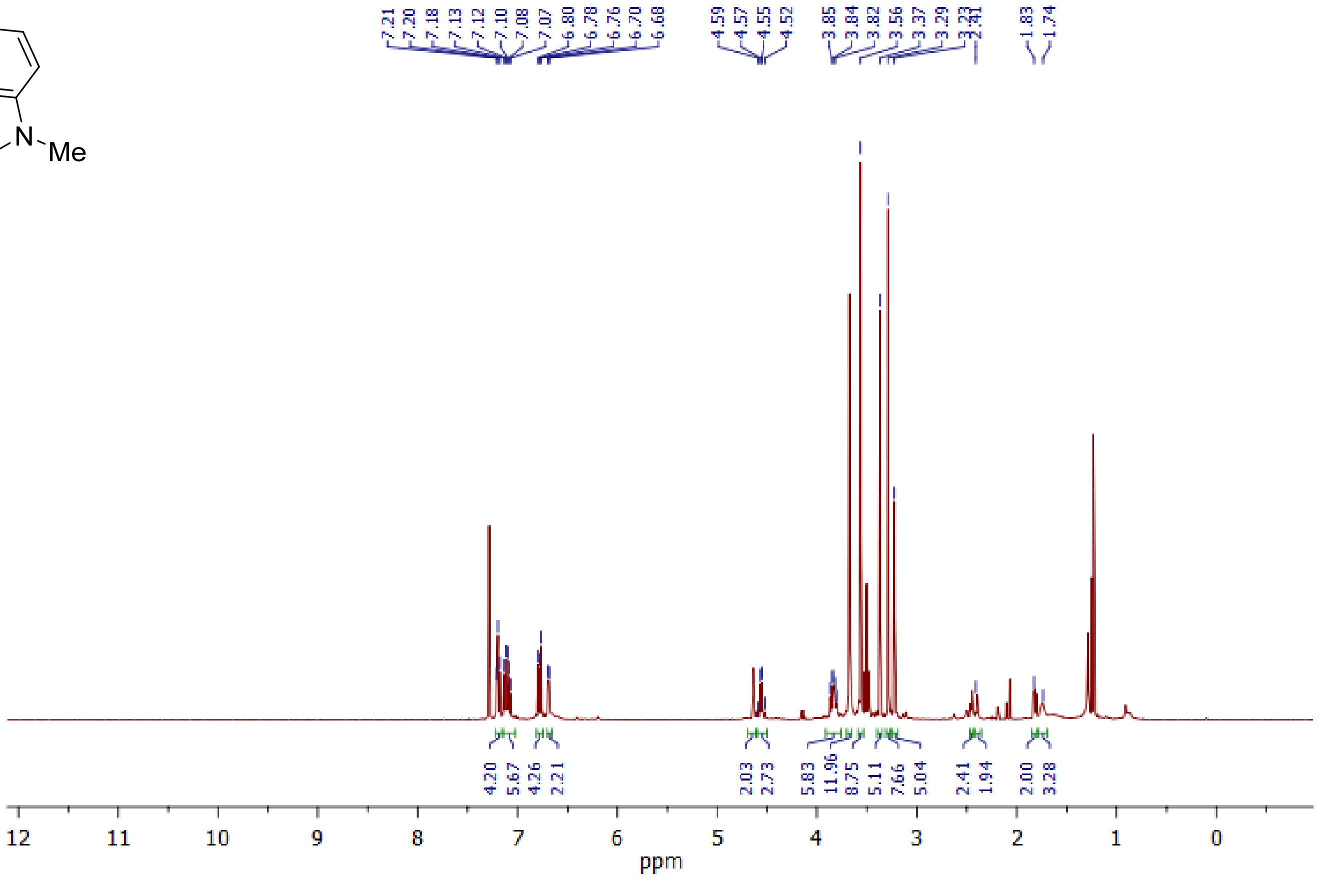

Dimethyl 2-[3-({4-[2,2-bis(methoxycarbonyl)cyclopropyl]-1-methyl-1*H*-indol-3-yl}methyl)-1-methyl-1*H*-indol-4-yl]cyclopropane-1,1-dicarboxylate (9)

^{13}C NMR ($CDCl_3$, 125 MHz)

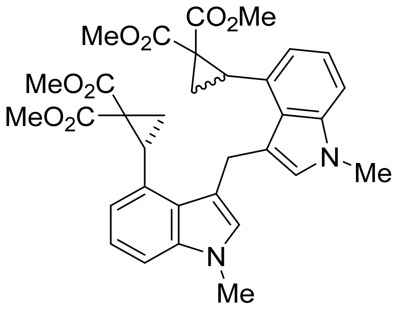

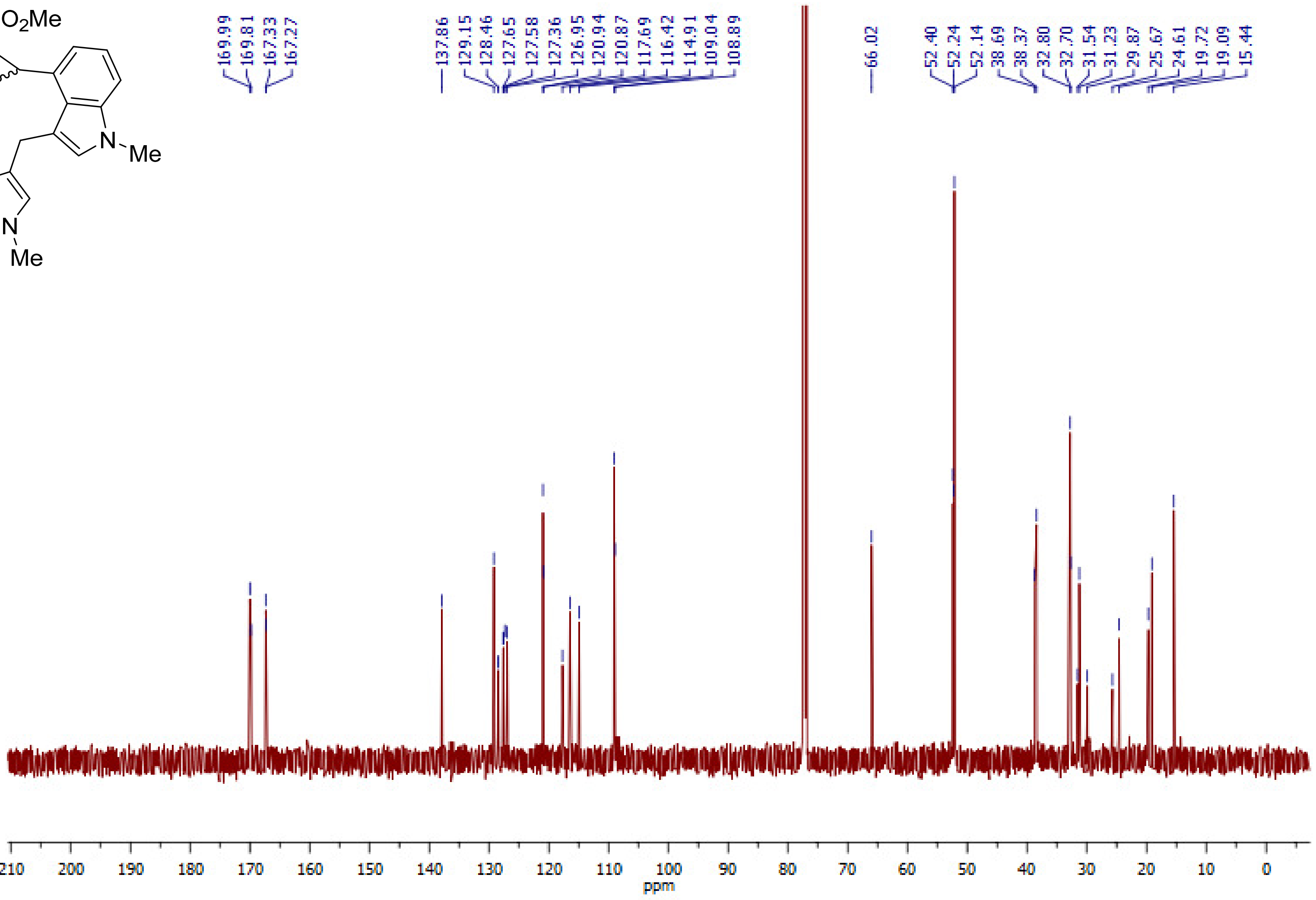

Dimethyl 2-[3-({4-[2,2-bis(methoxycarbonyl)cyclopropyl]-1-methyl-1*H*-indol-3-yl}methyl)-1-methyl-1*H*-indol-4-yl]cyclopropane-1,1-dicarboxylate (9)

HSQC ^{1}H-^{13}C ($CDCl_3$)

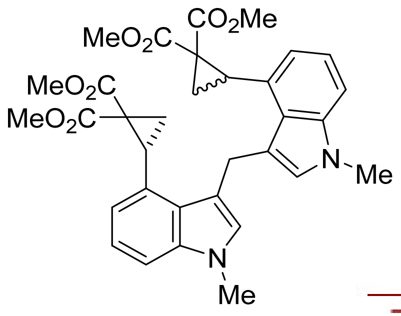

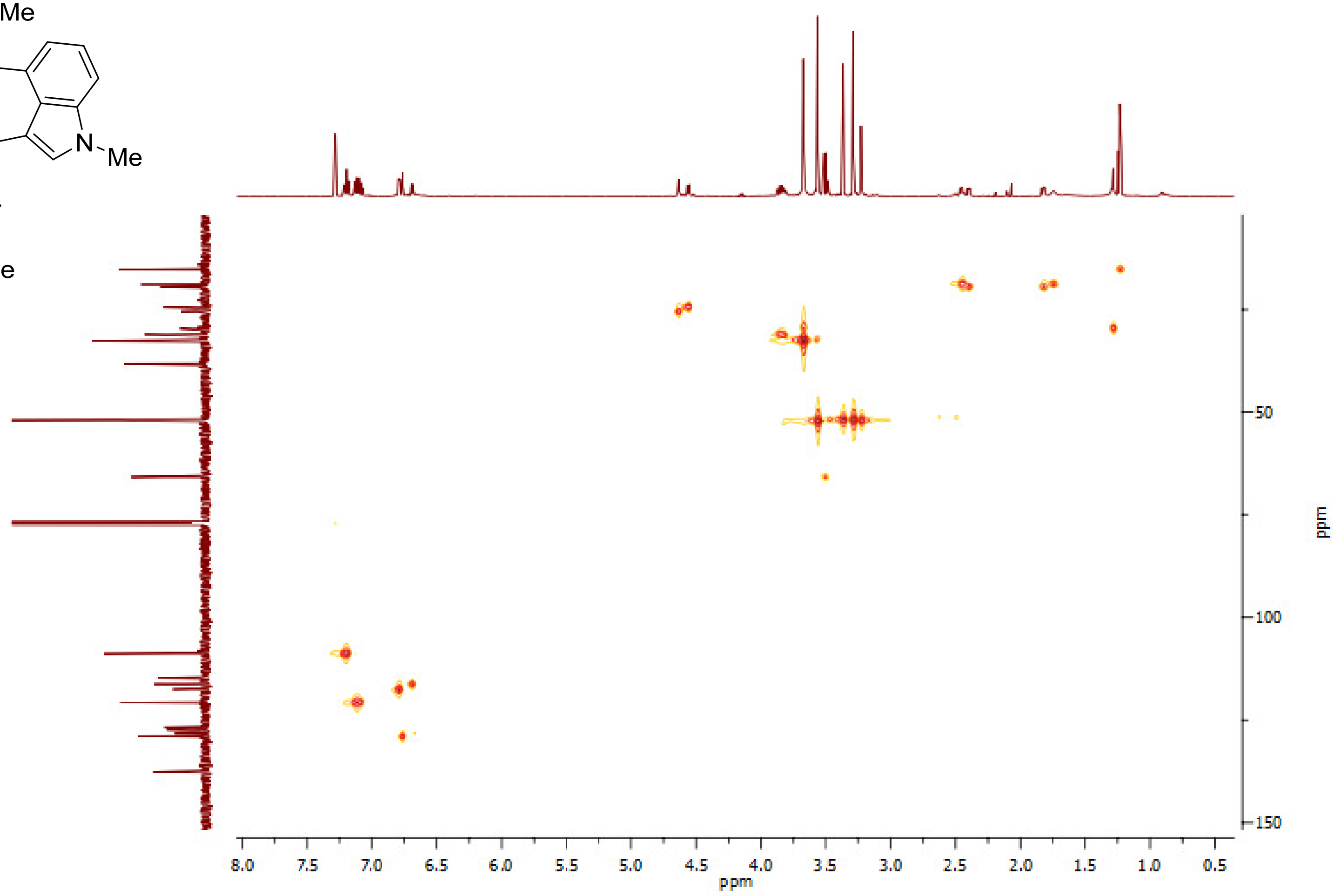

Dimethyl 2-[3-({4-[2,2-bis(methoxycarbonyl)cyclopropyl]-1-methyl-1*H*-indol-3-yl}methyl)-1-methyl-1*H*-indol-4-yl]cyclopropane-1,1-dicarboxylate (9)

HMBC ^{1}H-^{13}C ($CDCl_3$)

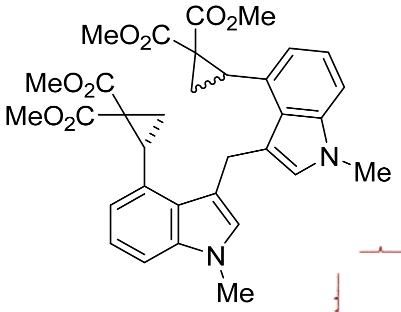

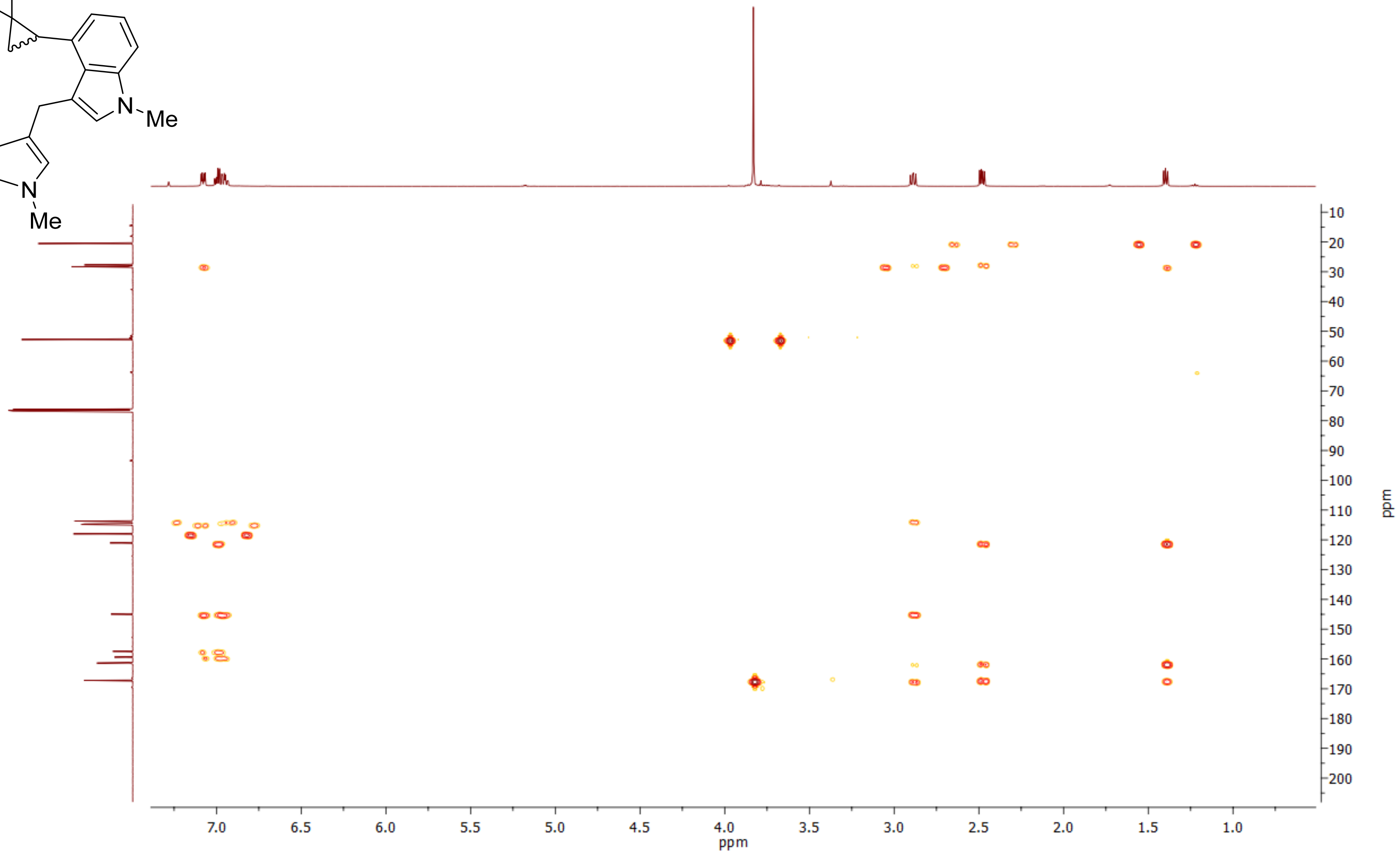

Dimethyl 2-[2-(5-fluoro-2-hydroxyphenyl)ethyl]malonate (10)

^{1}H NMR ($CDCl_3$, 400 MHz)

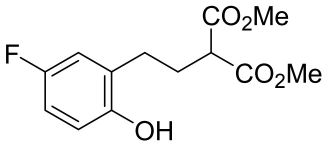

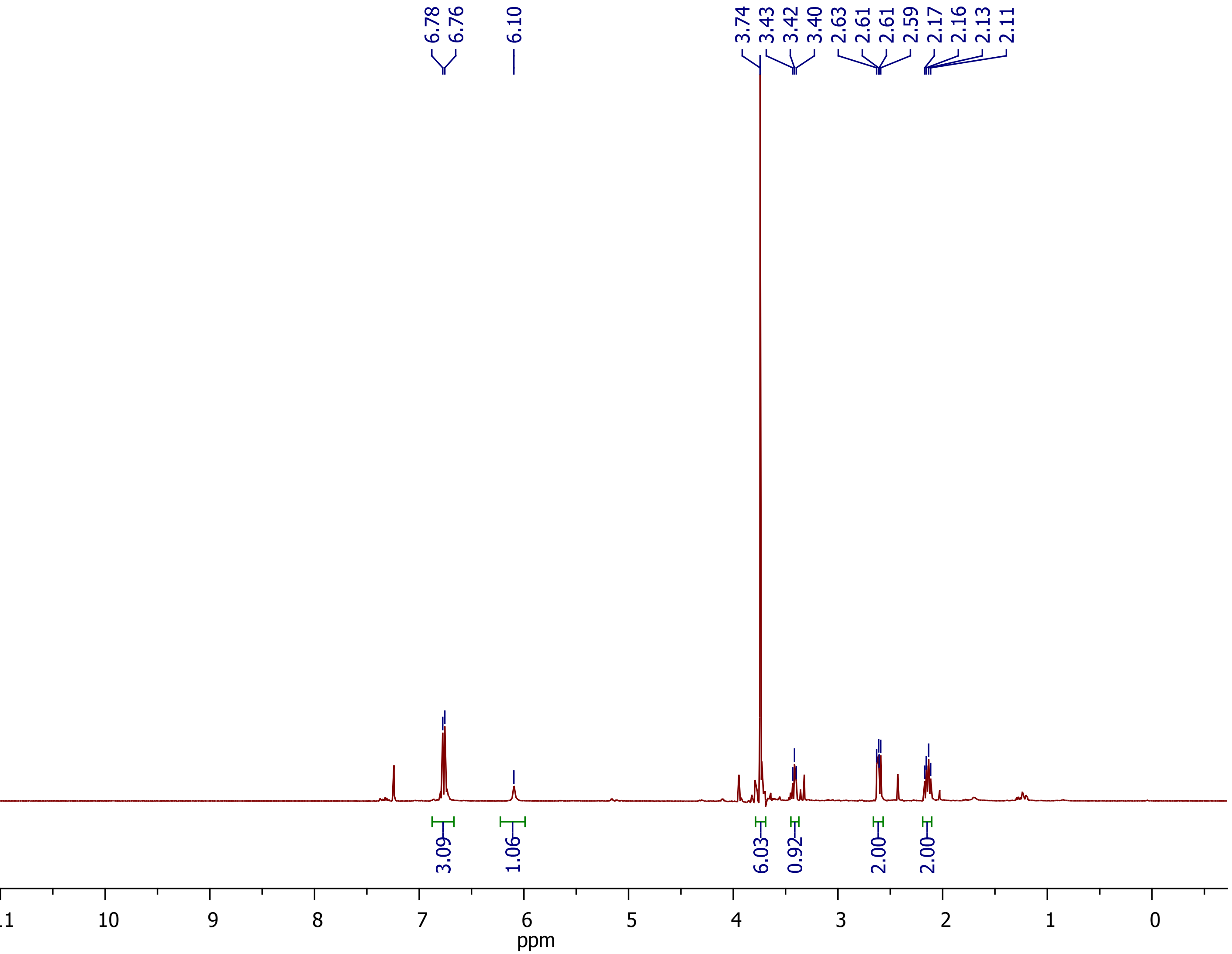

Dimethyl 2-[2-(5-fluoro-2-hydroxyphenyl)ethyl]malonate (10)

^{13}C NMR ($CDCl_3$, 100 MHz)

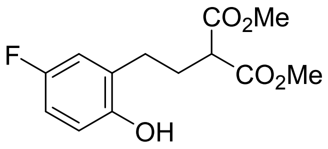

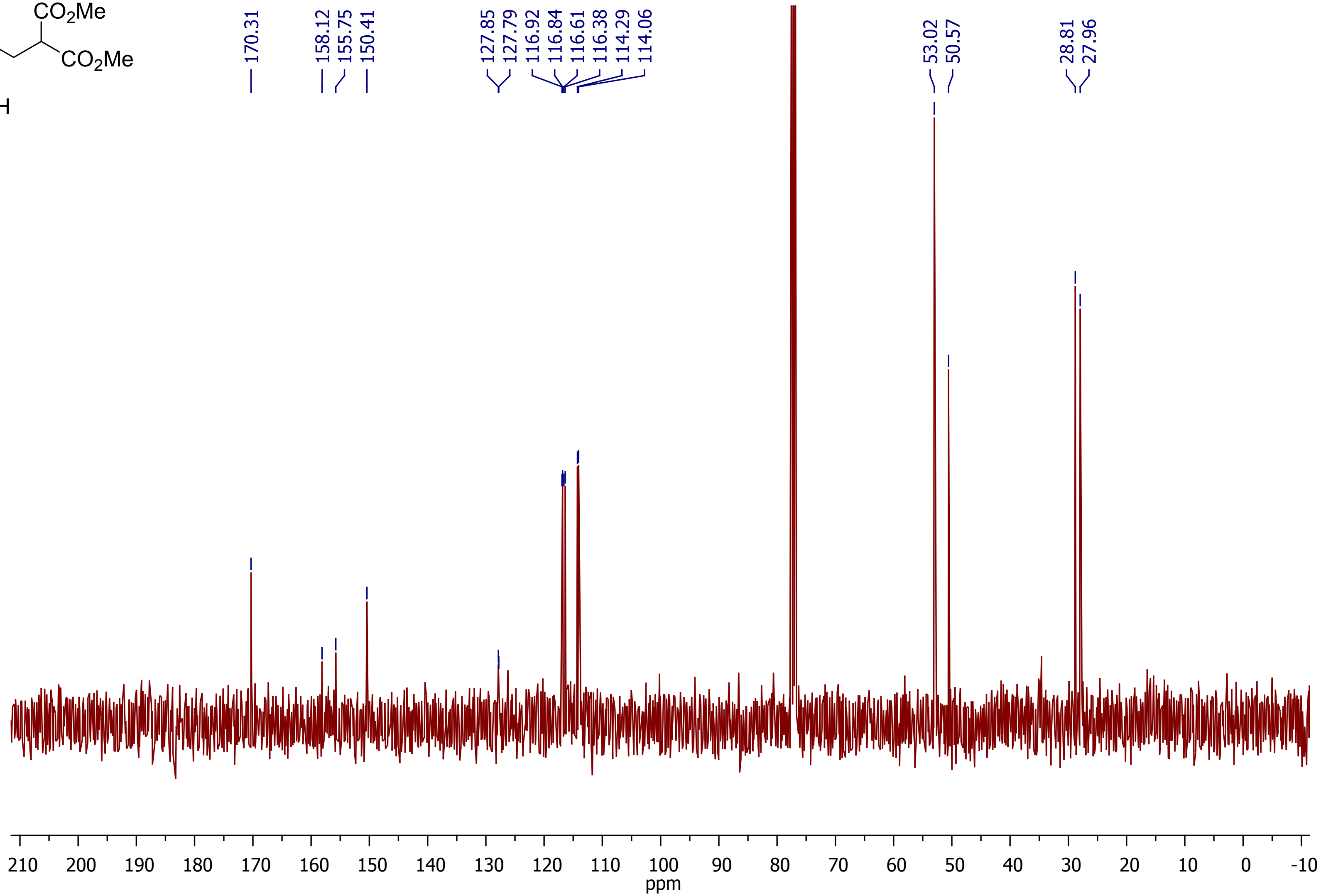

Supplement: Supplementary file 1 [file molecules-24-00057-s001.pdf]
